# Transcription factor RORα enforces stability of the Th17 cell effector program by binding to a *Rorc cis*-regulatory element

**DOI:** 10.1016/j.immuni.2022.09.013

**Published:** 2022-10-14

**Authors:** Jason A. Hall, Maria Pokrovskii, Lina Kroehling, Bo-Ram Kim, Seung Yong Kim, Lin Wu, June-Yong Lee, Dan R. Littman

**Affiliations:** 1. The Kimmel Center for Biology and Medicine of the Skirball Institute, New York University School of Medicine, New York, NY 10016, USA; 2. Department of Microbiology and Immunology, Yonsei University College of Medicine, Seoul, 03722, Korea; 3. Brain Korea 21 PLUS Project for Medical Sciences, Yonsei University College of Medicine, Seoul, 03722, Korea; 4. Institute for Immunology and Immunological Diseases, Yonsei University College of Medicine, Seoul, 03722, Korea; 5. Howard Hughes Medical Institute, New York, NY 10016, USA.; 6. These authors contributed equally; 7. Lead contact

**Keywords:** experimental autoimmune encephalomyelitis (EAE), autoimmunity, heat-labile enterotoxin, segmented filamentous bacteria (SFB), gene regulation

## Abstract

T helper 17 (Th17) cells regulate mucosal barrier defenses, but also promote multiple autoinflammatory diseases. Although many molecular determinants of Th17 cell differentiation have been elucidated, the transcriptional programs that sustain Th17 cells *in vivo* remain obscure. The transcription factor RORγt is critical for Th17 cell differentiation; however, it is not clear whether the closely-related RORα, which is co-expressed in Th17 cells, has a distinct role. Here, we demonstrated that, although dispensable for Th17 cell differentiation, RORα was necessary for optimal Th17 responses in peripheral tissues. The absence of RORα in T cells led to reductions in both RORγt expression and effector function amongst Th17 cells. Cooperative binding of RORα and RORγt to a previously unidentified *Rorc cis*-regulatory element was essential for Th17 lineage maintenance *in vivo*. These data point to a non- redundant role of RORα in Th17 lineage maintenance via reinforcement of the RORγt transcriptional program.

## eTOC blurb

The transcription factor RORα is recognized for contributing to Th17 cell differentiation and pathogenesis, but the underlying mechanisms are unclear. Hall, Pokrovskii et al. find that RORα reinforces the RORγt transcriptional program by binding to a *cis*- regulatory element within the *Rorc* locus that maintains RORγt expression *in vivo*, thus potentiating inflammatory disease.

## Graphical Abstract

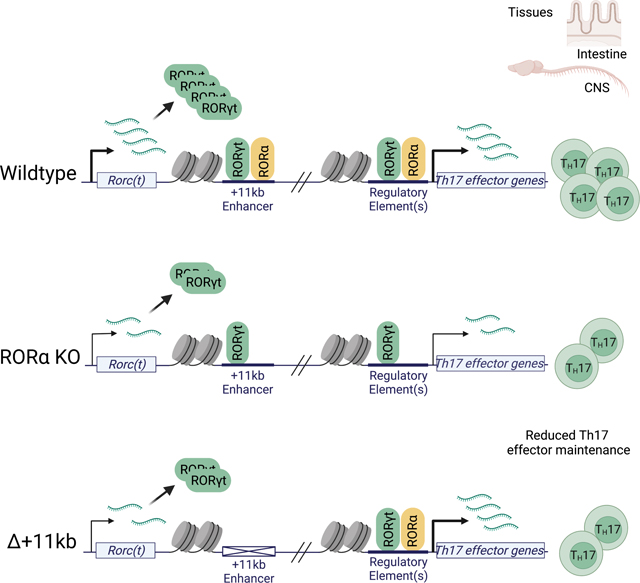

## Introduction

T-helper-17 (Th17) cells and related IL-17-producing (Type-17) lymphocytes are abundant at epithelial barrier sites (Honda and Littman, 2016). Their signature cytokines, IL-17A, IL-17F and IL-22, mediate an antimicrobial immune response and also contribute to wound healing and regeneration of injured tissues upon bacterial and fungal infection (Brockmann et al., 2017; Honda and Littman, 2016; Song et al., 2015). However, these cells are also key drivers of multiple chronic inflammatory diseases, including autoimmune diseases and inflammatory bowel disease (IBD), and they have also been implicated in carcinogenesis (Lee et al., 2020; Patel and Kuchroo, 2015; Stockinger and Omenetti, 2017). Ultimately, a better understanding of Type-17 regulatory mechanisms may uncover effective therapeutic strategies aimed at treating chronic inflammatory diseases and reducing cancer incidence.

The differentiation of Th17 cells and their ability to produce signature cytokines depend upon induction of the nuclear receptor (NR) transcription factor RAR-Related Orphan Receptor-gamma t (RORγt) (Ivanov et al., 2006). RORγt is required for the differentiation of both homeostatic Th17 cells, such as those that regulate commensal microbiota at mucosal barriers, and pro-inflammatory Th17 cells, whose dysregulation results in autoimmune and chronic inflammatory diseases. Therefore, identification of the context-dependent requirements for RORγt expression may facilitate understanding and therapeutic control of inflammatory immune responses. Studies conducted by our group and others have identified some of the *trans*-acting factors necessary for regulating transcription of *Rorc(t)* in Th17 cells (Ciofani et al., 2012; Durant et al., 2010; Schraml et al., 2009). However, the genomic *cis*-regulatory elements that control expression of RORγt in Th17 cells in vivo have been only partially characterized (Chang et al., 2020; Tanaka et al., 2014).

RORγt was initially described as the “master regulator” of the Th17 effector program (Ciofani et al., 2012; Ivanov et al., 2006; Ivanov et al., 2007; Miraldi et al., 2019). However, conditional deletion of *Rorc* (gene for RORγ and RORγt) in IL-17A-producing effector Th17 cells revealed RORγt to be essential for maintenance of Th17 cells, but not for development of immunopathology during experimental autoimmune encephalomyelitis (EAE) (Brucklacher-Waldert et al., 2016). Moreover, another ROR family transcription factor, RORα, is also upregulated during Th17 cell differentiation, can direct expression of IL-17 (Huh et al., 2011), and was reported to contribute to effector functions of Th17 cells and other related RORγt- expressing Type-17 lymphoid lineages (Castro et al., 2017; Fiancette et al., 2021; Stehle et al., 2021; Yang et al., 2008), suggesting that RORγt may not be solely responsible for the Th17 cell effector program. Our transcriptional regulatory network analysis of Th17 cells also identified RORα as a key Th17-promoting transcription factor (TF) (Ciofani et al., 2012; Miraldi et al., 2019).

In this study, we investigated the role of the closely-related RORα in regulating the Th17 effector program. By exploring the divergent effects of RORα and RORγt in Th17-driven autoimmune pathogenesis, we found that RORα is crucial for the functional maintenance of the Th17 program, despite exerting a relatively minor influence during differentiation of these cells. Thus, there was reduced accumulation of Th17 cells devoid of RORα in inflamed tissues, which manifested as a dampened pathogenic program. Analysis of chromatin occupancy and accessibility revealed that RORα binds to a *cis*-regulatory element within the *Rorc* locus and positively regulates RORγt expression during chronic autoimmune inflammation. Taken together, these findings suggest that RORα functions as a key regulator for the Th17 effector program through direct regulation of sustained RORγt expression during chronic inflammation.

## Results

### RORα and RORγt are differentially required for Th17-mediated EAE pathogenesis

**Figure 1.**
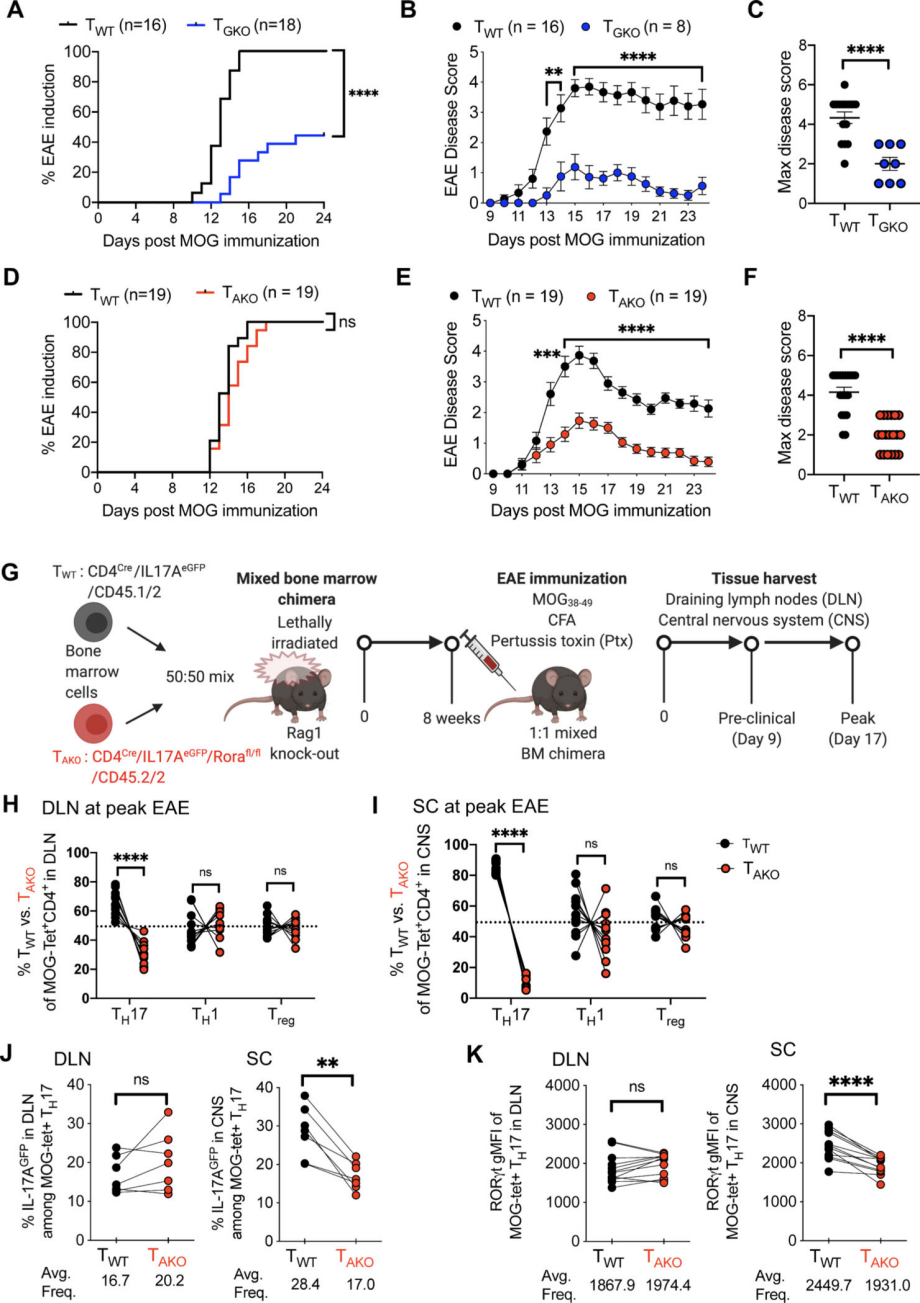
Divergent roles of RORγt and RORα in the differentiation and maintenance of pathogenic Th17 cells in autoimmune encephalomyelitis (EAE). **(A-C)** EAE frequency and severity in T cell-specific RORγt knock-out (T_GKO;_ *CD4*^Cre^*Rorc*^fl/fl^; n=18) and WT (*CD4*^Cre^; n=16) mice. Time course of EAE incidence **(A)** and mean daily disease score of symptomatic mice **(B)**; maximum disease score of EAE symptomatic mice **(C)**. Summary of 3 experiments. **(D-F)** EAE frequency and severity in T cell-specific RORα knock-out (T_AKO_; *CD4*^Cre^*Rora*^fl/fl^; n=19) and WT (*CD4*^Cre^; n=19), as in (A-C). Time course of EAE incidence **(D)** and mean daily disease score of symptomatic mice **(E)**; maximum disease score of EAE symptomatic mice **(F)**. Summary of 3 experiments. **(G)** Schematic of EAE induction in CD45.1/2 T_WT_ and CD45.2/2 T_AKO_ 50:50 (T_WT_/T_AKO_) mixed bone marrow (BM) chimeras. **(H and I)** Percent of T_WT_ and T_AKO_ cells of the indicated T cell phenotypes among MOG-tetramer+CD4^+^ T cells from draining lymph node (DLN; **H**) or spinal cord (SC; **I**) of T_WT_/T_AKO_ BM chimera at peak of EAE. Each phenotypic program was determined by the specific transcription factor expression by FACS (Th17: RORγt^+^FoxP3^Neg^CD44^hi^CD4^+^ T cells, Th1: T-bet^+^RORγt^Neg^ FoxP3^Neg^ CD44^hi^ CD4^+^ T cells, Treg: FoxP3^+^CD44^hi^CD4^+^ T cells). **(J)** Percent of *IL-17A*^eGFP+^ cells among MOG-tetramer^+^CD4+RORγt^+^ Th17 cells from DLN (left) or SC (right) of T_WT_/T_AKO_ BM chimera at peak of EAE. **(K)** RORγt gMFI (geometric mean fluorescence intensity) of MOG-tetramer^+^CD4^+^RORγt^+^ Th17 cells from DLN (left) or SC (right) of T_WT_/T_AKO_ BM chimera at peak of EAE. (A and D) Statistics were calculated by log-rank test using the Mantal-Cox method. (B and E) Statistics were calculated using the two-stage step-up method of Benjamini, Krieger and Yekutieliun. Error bars denote the mean ± s.e.m. (C and F) Statistics were calculated using the unpaired sample T test. Error bars denote the mean ± s.e.m. (E-I) Statistics were calculated using the paired sample T test. ns = not significant, *p < 0.05, **p < 0.01, ***p < 0.001, ****p < 0.0001. (H-K) Data combined from three experiments with 12 BM chimera mice. See also Figure S1.

Although it is established that RORγt is required for Th17 cell differentiation, it has been reported that RORα can partially compensate for RORγt deficiency to promote Th17-dependent experimental autoimmune encephalomyelitis (EAE) (Yang et al., 2008). To study whether these nuclear receptors exert distinct functions in Th17 cells, we studied mice harboring conditional deletions of *Rorc* and/or *Rora* in T cells. In line with previous studies, EAE disease was undetectable (10/18) or mild (8/18) in *CD4*^Cre^*Rorc*^fl/fl^ (T_GKO_) mice, compared to littermate *CD4*^Cre^*Rorc* wild-type (T_WT_) animals, which uniformly developed disease following immunization with myelin oligodendrocyte glycoprotein (MOG) in complete Freund’s adjuvant (CFA) and pertussis toxin (Ptx) injection (Figures 1A–C). To determine whether T_GKO_ cells were able to differentiate into Th17 cells in a setting permissive to fulminant EAE disease, we induced EAE in lethally-irradiated Rag1 deficient mice that had been reconstituted with an equal number of isotype-marked CD45.1/2 T_WT_ and CD45.2 T_GKO_ bone marrow cells. In this context, although all mice developed severe EAE, only cells of wild type origin were found to produce IL-17A in the draining lymph nodes (DLN) and spinal cord (SC). Conversely, the proportions of IFNγ-producing cells were similar among T_WT_ and T_GKO_ CD4^+^CD44^+^ T cells in DLN and SC, demonstrating that T_GKO_ cells retained the capacity to acquire effector functions (Figure S1A–C).

In contrast to T_GKO_ mice, mice with T cell-specific ablation of *Rora* (*CD4*^Cre^*Rora*^fl/fl^ (T_AKO_)) readily developed EAE (Figure 1D); however, disease severity was substantially milder than in control, littermate T_WT_ animals (Figures 1E and 1F). To probe the intrinsic role of RORα in pathogenic Th17 cell differentiation, we employed a 1:1 mixed bone marrow chimera strategy similar to that described above (Figures 1G and S1D). Notably, each donor strain also harbored an *Il17a*^eGFP^ reporter allele, in order to facilitate examination of myelin-specific Th17 cells using MOG-specific MHC class II (I-Ab-MOG_38–49_) tetramers (MOG-tet) (Figures 1G and S1E). Assessment in the DLN at the peak of EAE revealed a modest role for RORα in the differentiation of pathogenic Th17 cells, with an almost 2-fold reduction in the frequency of CD45.2/2 T_AKO_ effector Th17 (Foxp3^neg^RORγt^+^CD4^+^) cells relative to CD45.1/2 T_WT_ counterparts (Figures 1H and S1E). By contrast, the proportions of T-effector cells that exclusively expressed the Th1 lineage transcription factor, T-bet, or the regulatory T cell (Treg) lineage transcription factor, FoxP3, were roughly equivalent between the T_AKO_ and T_WT_ populations (Figures 1H and S1E). Notably, substantially more skewing (8.2-fold reduction) of the T_AKO_ population relative to wild-type cells was observed among RORγt^+^ Th17 cells in the SC (Figures 1I and S1F). Nevertheless, incorporation of the nucleoside analog EdU indicated that differentiating RORγt^+^ Th17 T_AKO_ effector cells proliferated similarly to their T_WT_ counterparts during the preclinical stage of disease (Figure S1G). Moreover, expression of the S-phase nuclear antigen, Ki67, remained similar in T_AKO_ and T_WT_-Th17 cells located in both the DLNs and SC throughout clinical stages of disease, suggesting that RORα does not regulate accumulation of Th17 cells in the SC via proliferation (Figures S1H and S1I). In concert with their lack of accumulation, MOG-tet+ Th17 T_AKO_ cells also exhibited signs of functional impairment in the SC, but not in the DLN, including reduction in proportion of cells expressing the *Il17a*^eGFP^ reporter and consistent decrease in the mean fluorescence intensity of RORγt expression (Figures 1J and 1K). These data suggest that while RORα is unable to mediate strong Th17 pathogenicity in the absence of RORγt expression, it maintains a prominent role in the regulation of the Th17 effector program.

### RORα is required for a sustained mucosal Th17 response

**Figure 2.**
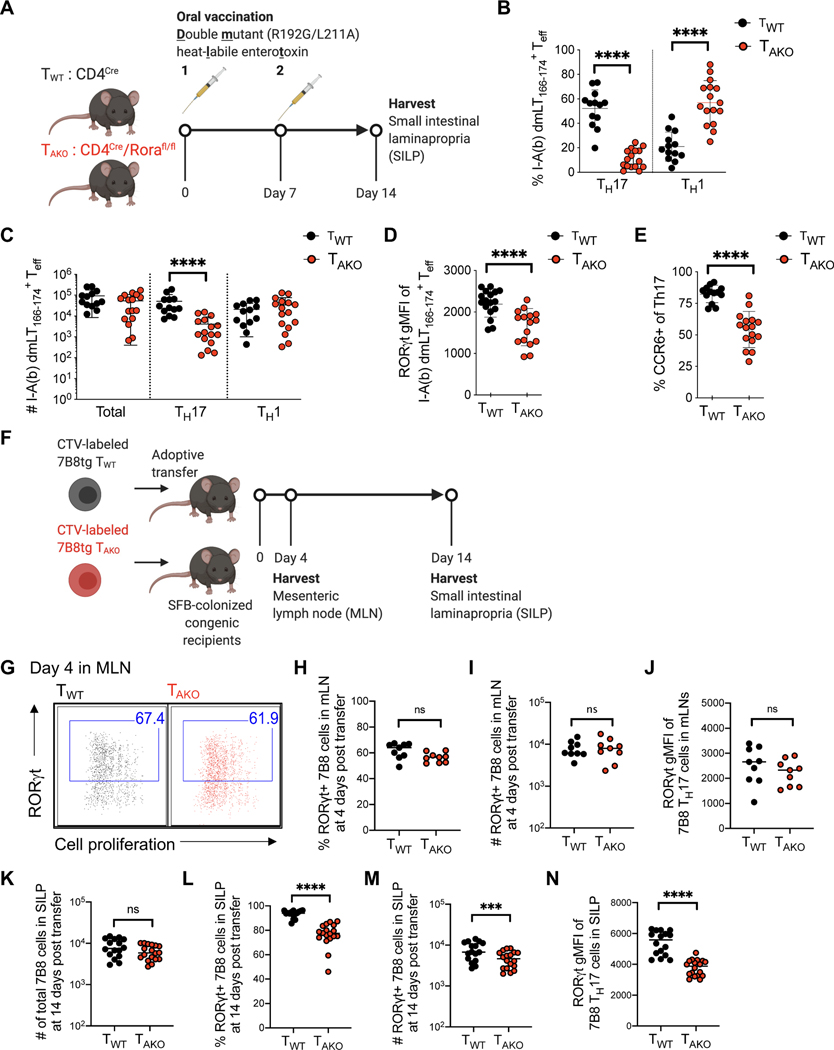
RORα drives sustained mucosal Th17 cell responses. **(A-E)** Oral vaccination of littermate T_WT_ and T_AKO_ mice with an attenuated double mutant (LT R192G/L211A) of the heat-labile enterotoxin of enterotoxigenic *Escherichia coli*, previously shown to induce a robust Th17 response. **(A)** Experimental scheme to examine the role of *Rora* in mucosal Th17 responses. **(B and C)** The proportion (B) and absolute number (C) of dmLT-specific Th17 and Th1 cells. Phenotypes were determined by FACS profiles for specific transcription factors (Th17: RORγt^+^FoxP3NegCD44hiCD4+ T cells, Th1: T-bet+RORγtNeg FoxP3Neg CD44hi CD4+ T cells, Treg: FoxP3+CD44hiCD4+ T cells). Data combined from three experiments with T_WT_ (n=13) and T_AKO_ (n=16) littermates. **(D)** RORγt gMFI of dmLT-specific Th17 cells. **(E)** Percentage of dmLT-specific Th17 cells expressing CCR6. **(F-N)** RORα deficiency impairs SFB-specific Th17 cell accumulation in SILP. **(F)** Experimental scheme to examine SFB-specific Th17 cell differentiation and effector function of 7B8tg T_WT_ and T_AKO_ in SFB-colonized hosts. **(G-J)** Characterization of donor-derived T_WT_ (n=9) and T_AKO_ (n=9) 7B8tg cells in recipients’ mesenteric lymph nodes (MLN) at 4 days post-adoptive transfer. Flow cytometric analysis of RORγt^+^ Th17 cell differentiation and expansion, monitored by Cell Trace Violet (CTV) dilution **(G)**, and frequency **(H)**, absolute number **(I)** and RORγt gMFI **(J)** of RORγt-expressing 7B8tg cells. Data combined from two experiments. **(K-N)** Characterization of donor-derived T_WT_ (n=16) and T_AKO_ (n=18) 7B8tg cells in recipients’ SILPs at 2 weeks post adoptive transfer. Summary of the total numbers **(K)** of SILP-accumulated 7B8tg cells, and frequency **(L)**, absolute number **(M)** and RORγt gMFI **(N)** of RORγt expressing 7B8tg cells. Data combined from three experiments. Statistics were calculated using the unpaired sample T test. Error bars denote the mean ± s.e.m. ns = not significant, *p < 0.05, ***p < 0.001, ****p < 0.0001. See also Figure S2.

To address whether the role of RORα in Th17 responses can be generalized, we orally vaccinated co-housed littermate T_WT_ and T_AKO_ mice with an attenuated double mutant (R192G/L211A) form of the heat-labile enterotoxin (dmLT) of enterotoxigenic *Escherichia coli*, which induces a robust antigen-specific mucosal Th17 response (Fonseca et al., 2015; Hall et al., 2008) (Figure 2A). Following two rounds of vaccination, dmLT-specific (I-Ab-dmLT_166–174_ tetramer positive) cells were readily detectable in the small intestinal lamina propria (SILP) of T_WT_ and T_AKO_ mice (Figure S2A). Yet, both the proportion and number of the dmLT-specific Th17 cells were substantially reduced in T_AKO_ mice (Figures 2B, 2C and S2B). Although this reduction was accompanied by a significant concomitant increase in the frequency of dmLT-specific Th1 cells within the SILP of T_AKO_ mice, both mutant and wildtype counterparts harbored similar numbers of dmLT-Th1 cells, suggesting that only the Th17 component of the effector T-cell response was impaired (Figures 2B, 2C and S2B). Amongst the dmLT-specific Th17 cells, the geometric mean fluorescence intensity (gMFI) of RORγt expression, as well as the frequency of RORγt^+^ cells that expressed CCR6, a RORγt-dependent chemokine receptor, were substantially reduced in T_AKO_ cells, reinforcing the notion that both RORα and RORγt are required to program and maintain optimal Th17 function (Figures 2D, 2E, S2C–E).

We additionally examined the role of RORα in the differentiation and maintenance of ileal homeostatic Th17 cells induced by segmented filamentous bacteria (SFB). This system allows for study of temporal regulation of Th17 cell differentiation, beginning with priming and proliferation in the draining mesenteric lymph node (MLN) and continuing with expansion and cytokine production in the lamina propria (Sano et al., 2015). T_WT_- or T_AKO_ mice were backcrossed with transgenic mice expressing a TCR (7B8tg) specific for a dominant epitope of SFB (Yang et al., 2014). Naïve 7B8tg T cells from these animals were labeled with Cell Trace Violet (CTV) and adoptively transferred into isotype-distinct hosts colonized with SFB (Figure 2F). Assessment of donor-derived T cells in the intestine-draining MLN revealed that CTV dilution and RORγt induction were similar between T_WT_ and T_AKO_ 7B8tg cells (Figures 2G–J), consistent with the notion that RORα is dispensable for commitment to the Th17 program. Accordingly, similar numbers of T_AKO_ and T_WT_ 7B8tg T cells were recovered two-weeks post-transfer from the terminal ileum section of the SILP, where SFB resides (Figure 2K). However, based on RORγt expression, there was a significant decrease in the proportion and total number of Th17 cells among T_AKO_ compared to T_WT_ 7B8tg T cells (Figures 2L, 2M and S2F), and the RORγt gMFI was also reduced in the mutant T cells (Figure 2N and S2G). Altogether, our results indicate that RORα confers the ability of T helper cells to mount a sustained Th17 cell response in target tissues.

### RORα is required for maintenance of the pathogenic Th17 program in the central nervous system

**Figure 3.**
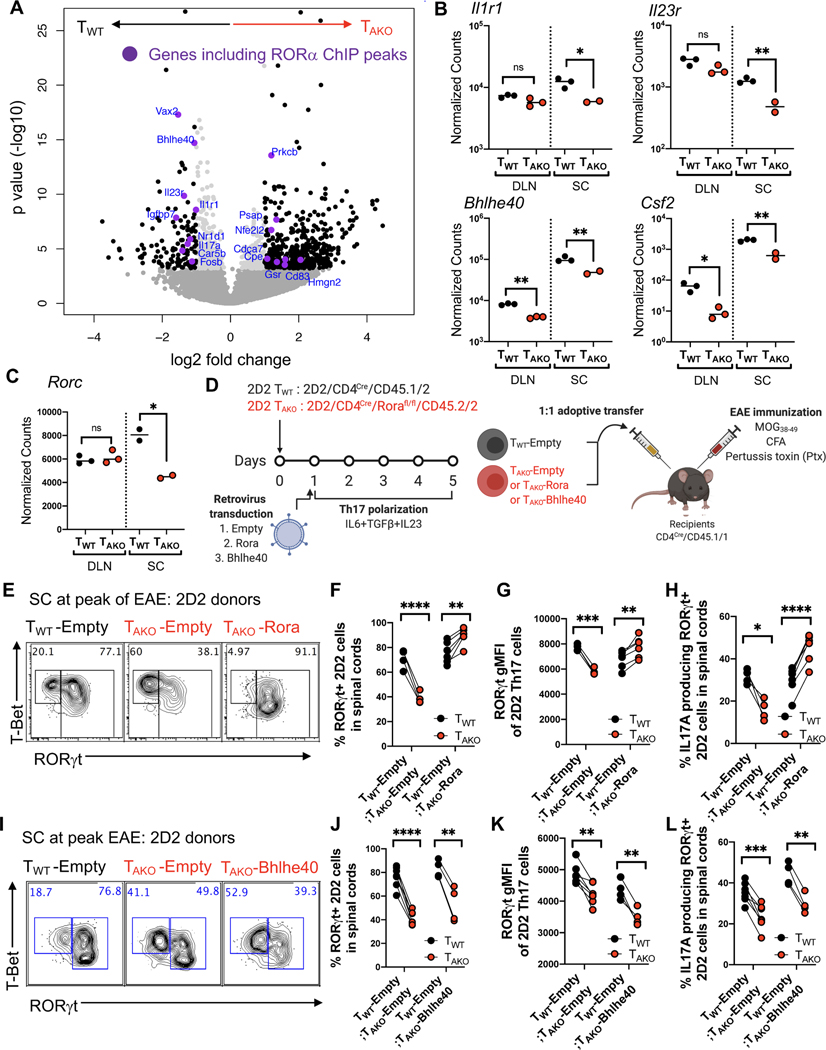
RORα stabilizes the Th17 transcriptional program in effector tissues. **(A-C)** RNA-Seq result of T_WT_ and T_AKO_ Th17 cells, isolated as *Il17a*^eGFP^-expressing T cells from the DLN and SC of 3 separate cohorts of mixed BM chimera mice at peak of EAE. **(A)** Volcano plot depicting differentially expressed (DE) genes of T_WT_ versus T_AKO_ *Il17a*^eGFP+^ Th17 cells from the SC. Black dots are significant DE genes. DE genes were calculated in DESeq2 using the Wald test with Benjamini-Hochberg correction to determine the false discovery rate (FDR < 0.01). Purple dots highlight genes that include RORα ChIP-Seq peaks within 10kb of the gene body. **(B and C)** Normalized counts of autoimmune disease-associated (*Il1r1, Il23r, Bhlhe40*), pathogenic (*Csf2*) genes **(B)** and *Rorc* **(C)** in T_WT_ and T_AKO_ *Il17a*^eGFP+^ Th17 cells from the DLN (T_WT_ (n = 3) and T_AKO_ (n = 3)) and SC (T_WT_ (n = 3) and T_AKO_ (n = 2)) at peak of EAE. Statistics were calculated using the unpaired sample T test. ns = not significant, *p < 0.05, **p < 0.01. **(D)** Experimental scheme to examine the role of RORα and BHLHE40 in maintenance of the auto-reactive effector Th17 program in inflamed SC during EAE. 2D2tg T_WT_ (*CD4*^Cre^/CD45.1/2) or T_AKO_ (*CD4*^Cre^/*Rora*^fl/fl^/CD45.2/2) cells were retrovirally transduced with *Rora* or *Bhlhe40* or control (Empty) vector, then in vitro polarized to Th17 cells (with IL-6+TGF-β+IL-23) for 5 days. The polarized T_WT_ and T_AKO_ 2D2 cells were combined 1:1 and transferred into recipients (CD4^Cre^/CD45.1/1) followed by EAE induction (MOG + CFA + Pertussis toxin immunization). **(E)** Flow cytometry analysis of RORγt and T-bet expression of T_WT_, *Rora*-deficient (T_AKO_ -Empty) and *Rora*-reconstituted (T_AKO_-Rora) 2D2 cells in SC at peak of EAE. **(F and G)** Frequency **(F)** and RORγt gMFI **(G)** of RORγt^+^ 2D2tg cells amongst donor T_AKO_-Empty or T_AKO_-Rora 2D2tg cells compared to the T_WT_-Empty in spinal cord at peak of EAE. **(H)** Frequency of indicated IL-17A-producing donor-derived 2D2tg-Th17 cells in SC at peak of EAE following *ex vivo* PMA/Ionomycin re-stimulation. **(I)** Flow cytometry analysis of RORγt and T-bet expression of T_WT_ –Empty and T_AKO_ -Empty or Bhlhe40 ectopic expressing (T_AKO_-Bhlhe40) cells in spinal cord at peak of EAE. **(J and K)** Frequency **(J)** and RORγt gMFI **(K)** of RORγt^+^ T_AKO_-Empty or T_AKO_–*Bhlhe40* 2D2 T_AKO_ cells compared to T_WT_ -Empty. **(L)** Frequency of indicated IL-17A-producing donor-derived 2D2tg-Th17 cells in SC at peak of EAE following *ex vivo* PMA/Ionomycin re-stimulation. (E-H) Summary of 2 experiments, with T_WT_-Empty:T_AKO_-Empty (n = 4) and T_WT_-Empty:T_AKO_-Rora (n = 6) recipients. Statistics were calculated using the paired sample T test. *p < 0.05, **p < 0.01, ***p < 0.001, ****p < 0.0001. (I-L) Summary of 2 experiments, with T_WT_-Empty:T_AKO_-Empty (n = 7) and T_WT_-Empty:T_AKO_-Bhlhe40 (n = 4) recipients. Statistics were calculated using the paired sample T test. *p < 0.05, **p < 0.01, ***p < 0.001, ****p < 0.0001. See also Figure S3.

To investigate the molecular mechanism by which RORα regulates the Th17 program, T_WT_ and T_AKO_ Th17 cells were isolated from the DLN and SC of 3 separate cohorts of mixed chimeric mice based on their IL17A^eGFP^ expression (see Figure 1G) at the peak of EAE disease, and their transcriptomes were sequenced (RNA-Seq) (Figures S3A–C). Based on the number of differentially expressed (DE) genes, *Rora* deficiency impacted the Th17 program more profoundly in the SC than in the DLN. At a false discovery rate of 1%, there were 33 DE genes in the DLN, but 845 genes in the SC (Figures 3A, S3B and S3C). At the peak of EAE, *Rora* mRNA expression in fully-committed Th17 cells within the spinal cord was also substantially higher than in differentiating precursors in the DLN (Figure S3D). These data further support a more prominent role for RORα in the regulation of Th17 cells within effector sites.

The most saliently affected gene in both differentiating (DLN) and effector (SC) T_AKO_-Th17 cells, *Bhlhe40*, was previously found to be required in both Th1 and Th17 cells for manifestation of EAE (Lin et al., 2016) (Figure 3B). T_AKO_-Th17 cells from the SC also exhibited significant reductions in transcripts encoding proteins that are prominent cell-intrinsic drivers of autoimmune pathogenesis, including *Csf2* (Codarri et al., 2011; El-Behi et al., 2011), *Il1r1* (Shouval et al., 2016), and *Il23r* (Abdollahi et al., 2016; Duerr et al., 2006; Gaffen et al., 2014; Hue et al., 2006) (Figure 3B). Indicative of the sweeping effect that loss of RORα engendered on gene expression at the site of disease, *Rorc*, which encodes RORγt, was markedly reduced in T_AKO_-Th17 cells from the SC, but not from DLN, consistent with reduced expression of direct RORγt target genes (Figure 3C). Meanwhile, the Th1 program genes, *Tbx21*, which encodes T-bet, and *Ifng* were not upregulated in T_AKO_-Th17 cells (Figure S3E). Thus, combined with the consistent, albeit modest, reduction in protein expression of RORγt in T_AKO_-Th17 cells at effector sites, including the SC and SILP (Figures 1K, 2D and 2N), these findings raise the possibility that RORα reinforces RORγt expression in effector Th17 cells.

To further explore this hypothesis, we developed a retroviral reconstitution system with T cells from MOG peptide-specific (2D2) TCR transgenic mice bred to RORα-deficient or wild-type mice. T_AKO_ 2D2 cells were transduced with *Rora* (yielding T_AKO_-Rora cells) or control (T_AKO_-Empty) vectors and were then cultured under Th17 cell differentiation conditions. They were then transferred with an equal number of similarly prepared isotype-marked T_WT_ 2D2 cells transduced with a control vector (T_WT_-Empty) into recipients that were subsequently immunized to induce EAE (Figure 3D). Critically, the *in vitro* differentiated T_AKO_-Rora, T_AKO_-Empty, and T_WT_-Empty 2D2 cells displayed uniform and equivalent expression of ROR*γ*t prior to adoptive transfer (Figure S3F). Yet, recapitulating the endogenous model, the frequency of RORγt^+^ cells amongst T_AKO_-Empty 2D2 cells in the SC at the peak of disease was markedly reduced relative to that of T_WT_-Empty 2D2tg cells (Figures 3E, 3F and S3G). Gating on the RORγt^+^ population also revealed a modest, though significant, decline in protein expression intensity, as well as an impaired capacity to produce IL-17A upon mitogenic restimulation (Figures 3G, 3H and S3H). Each of these deficits was reversed in T_AKO_-Rora 2D2tg cells, corroborating an essential role for RORα in maintenance of the Th17 effector program (Figures 3E–H, S3F–H). The pronounced effect of RORα on *Bhlhe40* expression in differentiating and effector Th17 cells suggested that it may influence Th17 stability indirectly, through BHLHE40, which is a critical regulator of autoreactive T cell pathogenicity (Lin et al., 2016; Lin et al., 2014). However, ectopic expression of *Bhlhe40*, despite rescuing impaired T_AKO_-2D2 cell accumulation (Figures S3I–K), failed to restore Th17 cell numbers or effector functions among 2D2-T_AKO_ cells (Figures 3I–L and S3L). Thus, regulation of *Bhlhe40* by RORα is not sufficient to direct effector Th17 cell maintenance, suggesting that RORα regulates other genes that are essential for this differentiation program.

### RORα shares genomic binding sites with RORγt

**Figure 4.**
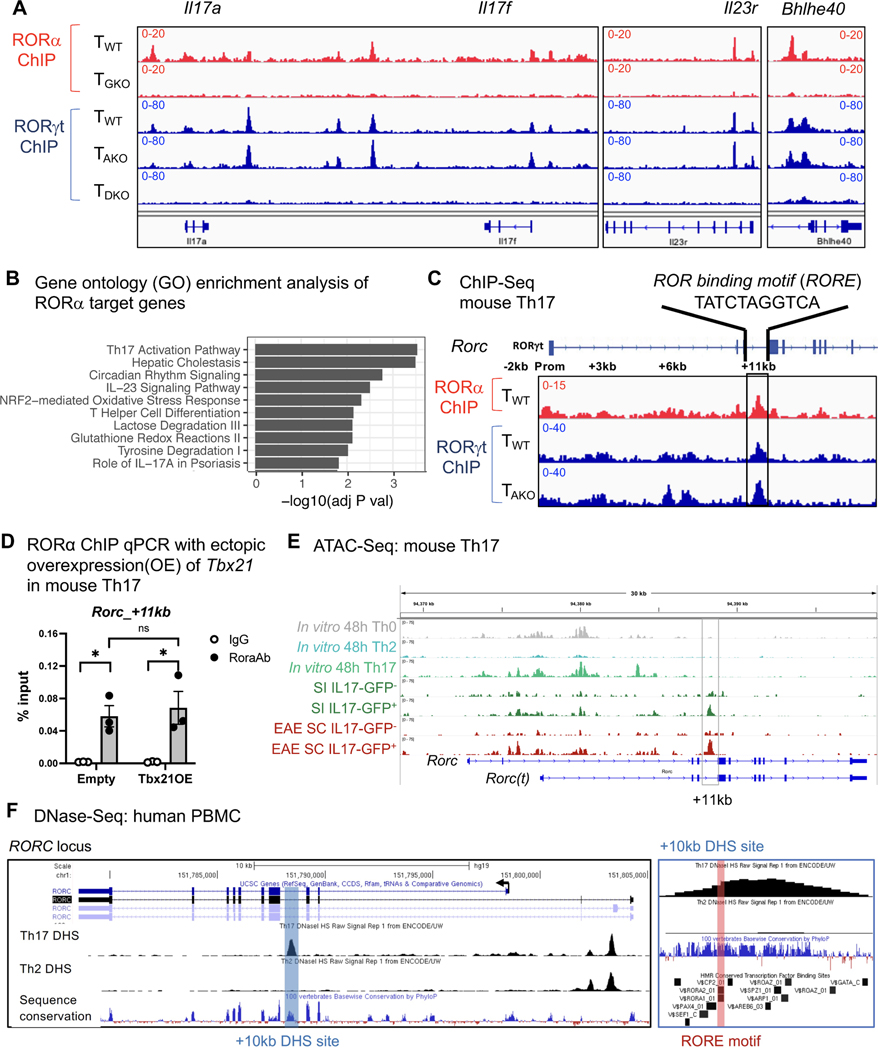
RORα shares genomic binding sites with RORγt in Th17 cells. **(A)** ChIP-Seq tracks of RORγt and RORα within Th17 effector program genes. **(B)** Gene ontology analysis of ROR*α* direct target genes (Peak(s) found within 10kb of gene body). **(C)** ChIP-Seq data exhibiting RORγt and RORα binding to cis-regulatory elements in *Rorc* locus. **(D)** RORα ChIP qPCR analysis of *the Rorc(t)* +11 kb locus with ectopic over-expression (OE) of *Tbx21* in *in vitro* polarized mouse Th17 cells, followed by chromatin immunoprecipitation with rabbit immunoglobulin G (IgG; control) or anti-RORα (Rora Ab) and quantitative PCR analysis of binding at the +11kb cis-regulatory element of *Rorc* (primers are listed in the STAR Methods). Results were normalized to those of a standardized aliquot of input chromatin. Summary of 3 experiments. Statistics were calculated using the paired sample T test. Error bars denote the mean ± s.e.m. ns = not significant, *p < 0.05 **(E)** ATAC-Seq data showing open cis-elements in the *Rorc* locus of *in vitro* differentiated or *ex vivo* isolated T cell lineages. Small intestine (SI) or EAE spinal cord (SC) T cells were FACS sorted from *Il17a*^eGFP^ mice gated on TCRβ+ then either GFP positive or negative. **(F)** UCSC genome browser depicting DNase-Seq on human Th17 (UCSC Accession: wgEncodeEH003020) and Th2 (UCSC Accession: wgEncodeEH000491) from the Encode database aligned with GRCh37/hg19 and the Vertebrate Multiz Alignment & Conservation (100 Species) and HMR Conserved Transcription Factor Binding Sites tracks. *RORC* locus (left) and zoomed +10kb DHS site (right). See also Figures S4 and S5.

To ascertain whether RORα directly regulates Th17 lineage maintenance, ChIP-Seq of RORα was performed with *in vitro* differentiated Th17 cells generated from RORα -Twin Strep (RORA-TS) tag knockin-in mice. These animals, which possess a Twin-Strep tag immediately upstream of the stop codon of the *Rora* locus, had normal development and immune cell functions, including frequencies of RORα-dependent type2 innate lymphoid cells (ILC2) (Figures S4A and S4B) and induction of both RORα and RORγt during *in vitro* Th17 cell differentiation on par with WT counterparts (Figures S4C and S4D). Alignment of RORα ChIP peaks with our previously published RORγt ChIP-Seq results for *in vitro* polarized Th17 cells (Ciofani et al., 2012) revealed substantial overlap of genome binding loci between RORα and RORγt, including previously reported genes involved in the “pathogenic” Th17 effector program (e.g., *Il17a/f*, *Il23r* and *Bhlhe40*) (Lee et al., 2012) (Figures 3A, 4A and S4E), and gene ontology analysis of the RORα direct target genes also revealed a significant enrichment in Th17 effector functions and Th17-mediated disease pathogenesis (Figure 4B). Notably, RORα also binds to intronic regions of *Rorc* (Figure 4C). To further address the interdependency of RORα and RORγt in binding to target loci, RORα ChIP-Seq was also conducted on Th17-polarized CD4+ T cells isolated from RORA-TS mice in which RORγt activity was abolished (RORA-TS-T_GKO_). Although loss of ROR*γ*t expectedly impeded Th17 cell differentiation (Figure S4F), both *Rora* induction and protein expression were comparable between WT and RORA-TS-T_GKO_ cells cultured under Th17 polarizing conditions (Figures S4G and S4H). Nevertheless, the majority of RORα peaks were ablated upon loss of RORγt (Figures 4A and S4E). In contrast, RORγt binding was not adversely affected in Th17-polarized cells that reciprocally lacked RORα (Figure S4I).

Reciprocal transcription factor networks containing RORα, RORγt, and T-bet were found to regulate the development of type 3 innate lymphoid cells (ILC3), such that deletion of T-bet rescues lymphoid tissue inducer (LTi) /ILC development in RORγt deficient animals/cells in a RORα-dependent manner (Fiancette et al., 2021; Stehle et al., 2021). Analogously, in helper T cells, although T_AKO_ cells did not exhibit numerical Th1 skewing in either pathological and homeostatic Th17 contexts, nor a Type-1 signature in committed T_AKO_ Th17 cells, we observed that additional deletion of T-bet rescued accumulation of 2D2 Th17 T_AKO_ cells in inflamed spinal cords at the peak of EAE (Figure S5A–E). This is consistent with previous data highlighting the ability of T-bet to antagonize RORγt expression through prevention of Runx1-transactivation of the *Rorc* promoter (Lazarevic et al., 2011). Conversely, ectopic expression of T-bet in *in vitro* polarized Th17 cells had no effect on RORα binding to key Th17-associated loci (*Il17a* and *Il23r* promoters) (Figure S5F–H). Moreover, T_AKO_ cells exhibited no enhanced T-bet expression after *in vitro* differentiation under Th17 polarizing conditions (Figure S5I). Thus, regulation of RORγt, not to mention the Th17 program, by T-bet and RORα likely occurs through autonomous mechanisms.

### The *Rorc(t)* +11kb locus is required for RORα-mediated RORγt expression in tissue-resident Th17 cells.

In support of the hypothesis that RORα can directly regulate RORγt expression, ChIP-Seq revealed a RORα peak co-localized with an embedded ROR response element (RORE) at +11kb from the *Rorc(t)* transcriptional start site in Th17 cells generated *in vitro* (Figure 4C). Alignment with RORγt ChIP-Seq data demonstrated that both family members bind to this region (Figure 4C). Similarly to other RORα genome binding loci implicated in the Th17 effector program (Figure S5F–H), T-bet had no effect on RORα binding to the *Rorc(t)* +11 kb *cis*-regulatory element locus (Figure 4D). Notably, although the assay for transposase-accessible chromatin sequencing (ATAC-Seq) indicated that this region remained closed in *in vitro*-differentiated Th17 cells, it was readily accessible in *ex vivo* IL-17A^+^ Th17 cells sorted from the SILP and SC during EAE (Figure 4E). Moreover, comparison of chromatin accessibility in T-helper lineages enriched from PBMC under the ENCODE Project (Maurano et al., 2012) revealed a prominent syntenic DNase Hypersensitivity Site (DHS) at +10kb from the *RORC* transcription start site (TSS) that was specific to Th17 cells, highlighting that this region constitutes a functionally conserved *cis*-regulatory element in human Type-17 immunity (Figure 4F). Altogether, these data suggest that synergy of RORα and RORγt binding to the intronic RORE following early RORγt induction governs subsequent RORγt stability in Th17 cells *in vivo*.

**Figure 5.**
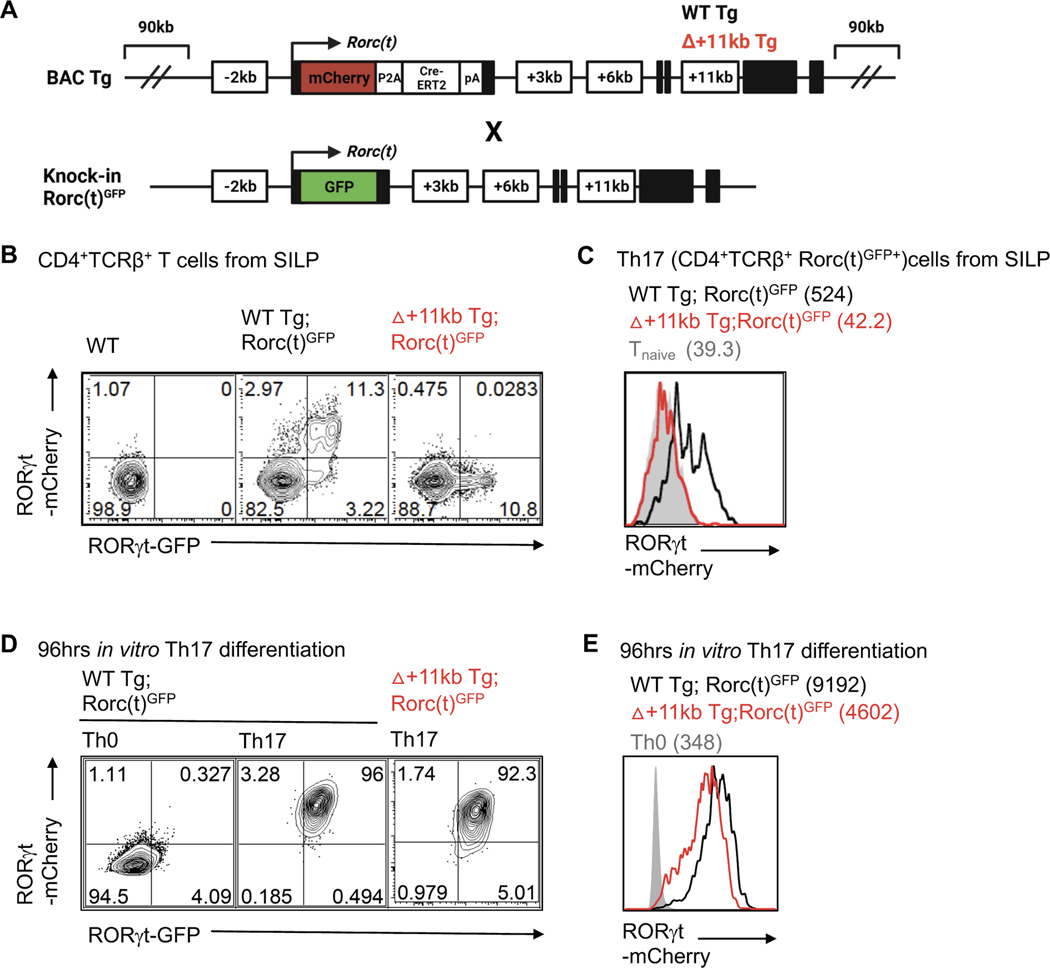
The *Rorc* +11kb cis-element is required for RORγt expression in Th17 cells *in vivo*, but is dispensable for *in vitro* differentiation. **(A)** Schematic depicting endogenous and BAC transgene allele in WT Tg (*Rorc(t)*-mCherry);*Rorc(t)*^GFP^ control or +11kb cis-regulatory element mutant (Δ+11kb) Tg (Δ+11kb *Rorc(t)*-mCherry);*Rorc(t)*^GFP^ mice. **(B and C)** Flow cytometry plots (B) and stacked histogram (C) illustrates RORγt-mCherry reporter expression in *ex vivo* isolated Th17 (TCRβ^+^RORγt^GFP+^) cells from SILP of WT Tg (*Rorc(t)*-mCherry);*Rorc(t)*^GFP^ control or +11kb cis-regulatory element mutant (Δ+11kb) Tg (Δ+11kb *Rorc(t)*-mCherry);*Rorc(t)*^GFP^ mice. gMFIs are included in parentheses. Representative data of three experiments. **(D and E)** Flow cytometry plots (D) and stacked histogram (E) illustrate RORγt-mCherry reporter expression in *in vitro* differentiated Th17 cells from WT Tg;*Rorc(t)*^GFP^ or △+11kbTg;*Rorc(t)*^GFP^ mice. gMFI are included in parentheses. Representative data of three experiments. See also Figure S6.

To functionally interrogate the role of the *Rorc(t)* +11kb *cis*-regulatory element *in vivo*, we generated transgenic mice with a *Rorc*-containing BAC engineered to have a mCherry reporter at the RORγt translational start site with or without deletion of the +11kb *cis*-regulatory element (WT Tg (Rorc(t)-mCherry and Δ+11kb Rorc(t)-mCherry) (Figure 5A). To serve as an internal control, the transgenic mice were bred to *Rorc(t)*^eGFP^ mice containing a GFP reporter knocked into the endogenous *Rorc(t)* locus (Eberl and Littman, 2004) (Figure 5A and S6A). Thymocyte development was normal in both WT Tg and Δ+11kb Tg lines, with mCherry expression highest in double positive and early post-selection single positive thymocytes, consistent with known expression patterns of RORγt (He et al., 2000; Sun et al., 2000) (Figure S6B). Within the SILP, a strong correlation between GFP and mCherry expression was also observed in both innate and adaptive Type-17 lymphocytes, which included not only Th17 cells, but also γδT cells and type 3 innate lymphoid cells (ILC3) of WT Tg mice (Figure 5B, 5C and S6C–E). In stark contrast, mCherry activity was lost in each of these SILP lymphocyte populations in Δ+11kb Tg mice, suggesting that the +11kb *cis*-regulatory element is a bona fide enhancer for all Type-17 lymphocyte lineages in vivo (Figure 5B, 5C and S6C–E). Nevertheless, CD4+ T cells isolated from Δ+11kb Tg mice readily expressed mCherry upon *in vitro* Th17 polarization (Figure 5D and 5E). This finding, together with the chromatin accessibility data for *in vitro* polarized Th17 cells (Figure 4E), as illustrated by failure to open chromatin at the +11kb locus, suggests that the +11kb *cis*-regulatory element is an essential enhancer for the Type-17 lymphocytes *in vivo* but is dispensable for thymocyte development and *in vitro* Th17 cell differentiation.

**Figure 6.**
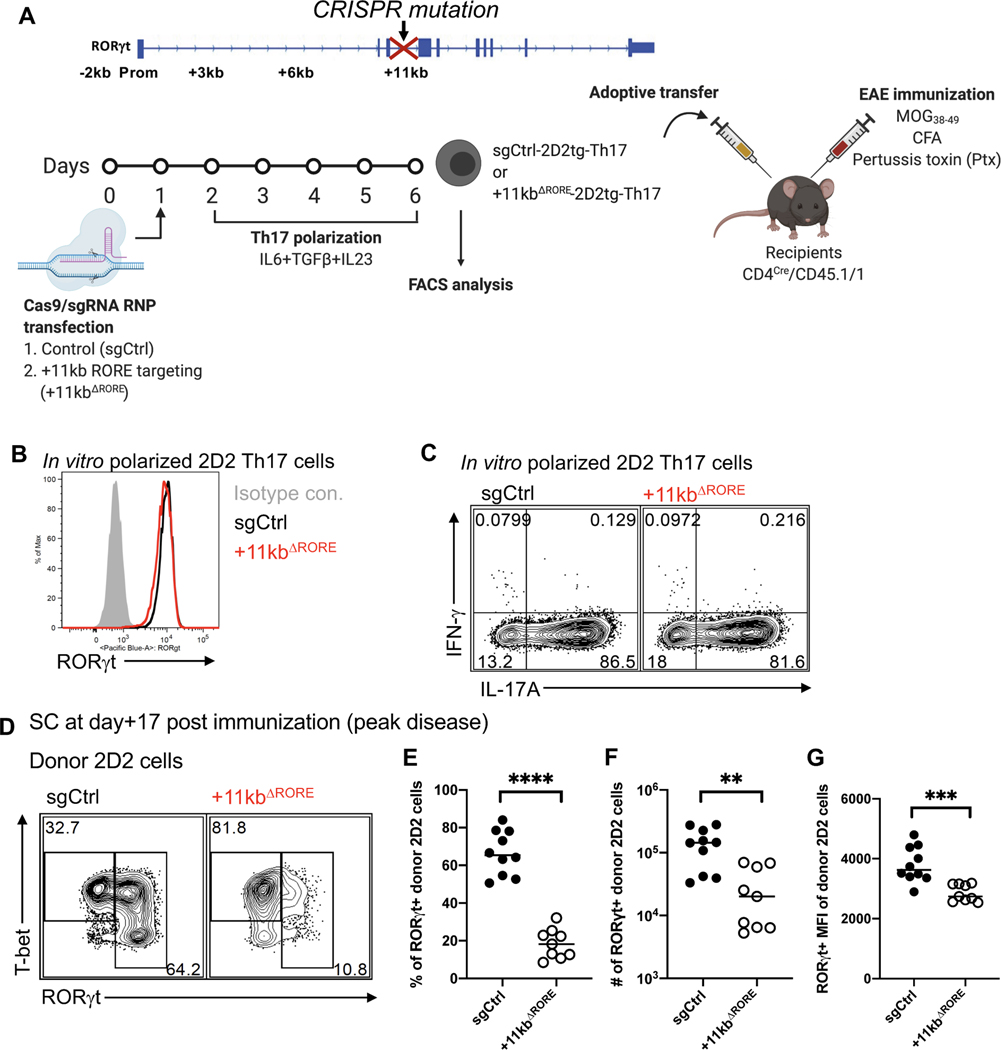
The *Rorc(t)* +11kb locus is required for maintenance of RORγt expression in tissue-resident Th17 cells. **(A)** Experimental scheme to interrogate the role of the *Rorc(t)* +11kb element *in vivo.* **(B)** Stacked histogram illustrates RORγt expression in control (sgRNA control; sgCtrl) and *Rorc(t)* +11kb cis-regulatory element mutant (sgRNA that target RORE in +11kb cis-element of *Rorc(t)*; +11kb^ΔRORE^) *in vitro* differentiated 2D2tg Th17 cells. **(C)** Representative FACS plots displaying IL-17A and IFNγ production of *in vitro* polarized Th17 sgCtrl or +11kb^ΔRORE^ 2D2tg cells. **(D)** Representative flow cytometry analysis of RORγt and T-bet expression in sgCtrl and +11kb^ΔRORE^ donor-derived 2D2tg cells in SC at peak of EAE. **(E-G)** Frequency (E), number (F) and RORγt gMFI (G) of RORγt-expressing sgCtrl or +11kb^ΔRORE^ 2D2tg cells in SC at peak of EAE. Summary of 2 experiments, with sgCtrl (n = 10) and +11kb^ΔRORE^ (n = 9) recipients. Statistics were calculated using the unpaired sample T test. Error bars denote the mean ± s.e.m. ns = not significant, *p < 0.05, **p < 0.01, ***p < 0.001, ****p < 0.0001. See also Figure S7.

**Figure 7.**
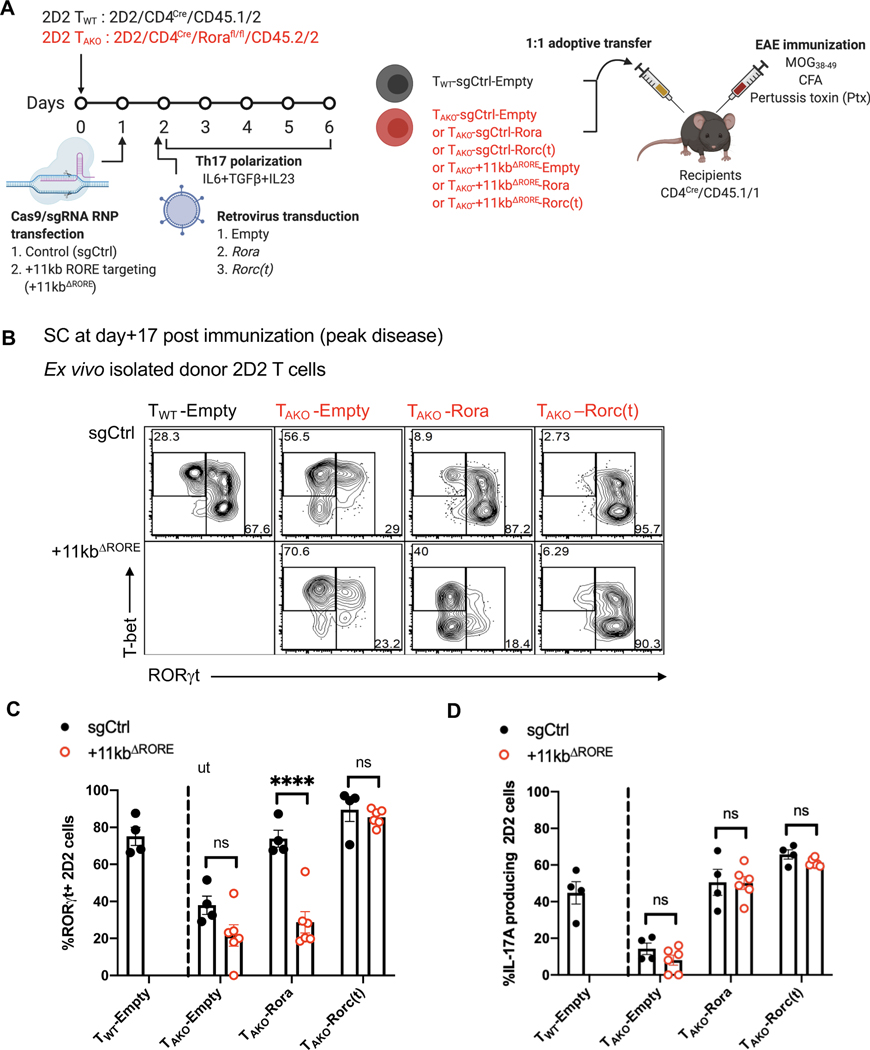
RORα promotes *in vivo* Th17 stability through a conserved *cis*-regulatory element located in the +11kb region of the *Rorc(t)* locus. **(A)** Experimental scheme to examine the role of *Rorc(t)* +11kb *cis*-regulatory element in maintenance of the pathogenic Th17 program during EAE. **(B and C)** Flow cytometry analysis of RORγt and T-bet expression (B) and frequency of RORγt expression (C) in sgCtrl or +11kb^ΔRORE^ T_AKO_ donor-derived 2D2tg cells, retrovirally reconstituted with *Rora* or *Rorgt*, in SC at peak of EAE. Summary of 2 experiments with following cell combinations: T_WT_-sgCtrl-Empty:T_AKO_-sgCtrl-Empty (n=4), T_WT_-sgCtrl-Empty:T_AKO_-sgCtrl-Rora (n=4), T_WT_-sgCtrl-Empty:T_AKO_-sgCtrl-Roc(t) (n=4), T_WT_-sgCtrl-Empty:T_AKO_-*+11kb*^ΔRORE^ -Empty (n=5), T_WT_-sgCtrl-Empty:T_AKO_-*+11kb*^ΔRORE^-Rora (n=5), T_WT_-sgCtrl-Empty:T_AKO_-*+11kb*^ΔRORE^ -Roc(t) (n=5) recipients. **(D)** Frequency of IL-17A production among sgCtrl or +11kb^ΔRORE^ T_AKO_ donor-derived 2D2tg cells, retrovirally reconstituted with *Rora* or *Rorc(t)*, in SC at peak of EAE. Summary of 2 experiments. Statistics were calculated using the unpaired sample T test. Error bars denote the mean ± s.e.m. ns = not significant, *p < 0.05, **p < 0.01, ***p < 0.001, ****p < 0.0001. See also Figure S7.

To further investigate whether the +11kb conserved noncoding sequence functions via the binding of ROR family TFs in EAE, an optimized Cas9/gRNA RNP transfection approach was utilized to mutate the RORE and preclude RORα and ROR*γ*t binding to the +11kb *cis*-regulatory element in *in vitro*-differentiated Th17 cells (Figure 6A). Targeting the locus in activated naïve 2D2tg-T cells resulted in nearly 100% editing efficiency, with both indels and deletions that did not exceed 100bps (Figures S7A and S7B). Following *in vitro* Th17 cell polarization with IL-6, TGF-β and IL-23, control gene (sgCtrl) and +11kb-*cis*-regulatory element-targeted (+11kb^ΔRORE^) 2D2tg-Th17 cells were adoptively transferred into wild-type recipients, which were then immunized with MOG peptide to trigger EAE (Figure 6A). Consistent with the inaccessibility the *Rorc* +11kb site in *in vitro* polarized Th17 cells, neither the induction of RORγt, nor the capacity to secrete IL-17A, were affected in the +11kb^ΔRORE^ 2D2tg-Th17 cells at the time of transfer (Figures 6B and 6C). However, by the peak of disease, in comparison to control-targeted counterparts, both the percentage and absolute number of RORγt^+^ +11kb^ΔRORE^ 2D2tg-Th17 cells recovered from the SC sharply declined (Figures 6D–F). Among the residual Th17 cells, RORγt expression was also markedly reduced (Figure 6G). These findings reflect the compromised maintenance of RORγt expression in T_AKO_ 2D2tg-Th17 cells during EAE (Figures 3E–H). Accordingly, ectopic overexpression of *Rora* restored RORγt expression in T_AKO_ 2D2tg-Th17 cells, but not in T_AKO_ +11kb^ΔRORE^ 2D2tg-Th17 cells (Figures 7A–C), consistent with RORα binding to the +11kb *cis*-regulatory element mediating sustained expression of RORγt. In contrast, ectopic overexpression of *Rora* did fully rescue IL-17A production (Figure 7D). These data suggest that RORα not only regulates the Th17 program in a similar way to RORγt, but may also play a key role *in vivo* by dynamically reinforcing RORγt expression in the absence of saturating amounts of active RORγt. Thus, our findings uncover a previously unidentified *cis*-regulatory element required for maintenance of the Th17 cell program in tissues and regulated, at least in part, by RORα.

## Discussion

Our current study confirms that both RORα and RORγt play important roles in orchestrating Th17 lineage maintenance. Our data suggest that RORα and RORγt may regulate the expression of Th17-associated genes through binding to the same ROREs with their highly similar DNA-binding domains (Cook et al., 2015). This implies that the individual expression of RORα and RORγt might be limiting in T cells, leaving ROREs unoccupied, and that expression of both nuclear receptors is required to saturate RORE binding sites and drive maximal ROR-responsive gene expression. We also observed that expression of RORγt is a prerequisite for RORα binding to the shared RORE. In the absence of RORγt, the T_GKO_ Th17 cells lost most of the genome-wide binding of RORα at the shared target sites. Considering that RORα expression was not impaired upon RORγt deletion, these data are consistent with the model previously proposed by Ciofani et. al, in which RORγt serves as a master switch for Th17 differentiation and creates a feedback pathway that, in turn, stabilizes Th17 commitment (Ciofani et al., 2012).

Another possible scenario is that RORα and RORγt may bind to DNA cooperatively. Like all nuclear receptors, ROR proteins have been shown to bind cognate DNA elements as monomers or dimers: as monomers to ROREs containing a single consensus half site (PuGGTCA) immediately preceded by a short A/T-rich region, and as dimers to tandem half sites oriented as palindromes, inverted palindromes, or direct repeats (Giguere, 1999). Indeed, RORα:RORγt heterodimers could possess distinct functional activity compared to monomers or homodimers because of their distinct N-terminal trans-activation domains (NTDs) (Giguere, 1999; McBroom et al., 1995).

Chronic inflammation underlies a number of debilitating human diseases including inflammatory bowel disease, multiple sclerosis, psoriasis, and various arthritides (Bamias et al., 2016; Firestein and McInnes, 2017; Netea et al., 2017; Noda et al., 2015). Th17 cells have central roles in many of these diseases. The transcription factor RORγt was initially coined the master regulator of the Th17 program, but targeting RORγt therapeutically is dangerous owing to an enhanced risk of thymoma upon its inhibition (Guntermann et al., 2017; Guo et al., 2016; Liljevald et al., 2016). RORα was also implicated in Th17 functions (Castro et al., 2017; Yang et al., 2008), and pharmacological blockade of RORα has been reported to suppress EAE (Wang et al., 2021), but its precise role and relationship to RORγt function were not investigated. Exploration of the divergent effects of RORα and RORγt in Th17-elicited autoimmune pathogenesis revealed that RORα is crucial for the functional maintenance of the Th17 program at the site of inflammation despite exerting a relatively minor influence during differentiation in the lymph nodes. During EAE, Th17 cells devoid of RORα were limited in their accumulation in the central nervous system, and those that present displayed a dampened pathogenic program. Probing the intersection of ROR binding targets identified by ChIP-Seq with RNA-Seq data obtained from *ex vivo* isolated RORα-deficient Th17 cells indicated that the majority of RORα targets are shared with ROR*γ*t. Among the most significant were the IL-23 receptor, *Il23r*, and the transcription factor *Bhlhe40*, which are critical for driving Th17 pathogenesis by way of inflammatory T cells having shared Th17 and Th1 features (Harbour et al., 2015; Hirota et al., 2011). Notably, RORα was also found bound to a conserved *cis*-regulatory element in the *Rorc* locus that is crucial for maintenance of RORγt expression in effector Th17 cells *in vivo*. Using our laboratory’s previous transcription factor binding data (Ciofani et al., 2012), Chang et al. recently identified this region (CNS11) in their study of Th17 enhancers, but did not prosecute its function owing to its lack of H3K27Ac marks and weak interaction with p300 (Chang et al., 2020). These data are also consistent with the marginal chromatin accessibility of the +11kb region observed upon *in vitro* differentiation and suggests that a heretofore unidentified factor mediates *in vivo* accessibility of this region.

Natural ligands and synthetic compounds that modulate the function of nuclear receptors have demonstrated tremendous therapeutic potential for multiple clinical conditions (Cheng et al., 2019; Huh et al., 2011; Kojetin and Burris, 2014; Marciano et al., 2014; Moutinho et al., 2019). Our current study, by identifying RORα as a key regulator of the sustained Th17 effector program, suggests that targeting this receptor could be a viable strategy for treating autoimmune pathologies linked to Th17 effector functions in chronically inflamed patient tissues. Furthermore, the involvement of RORα in ILC2 development (Halim et al., 2012; Wong et al., 2012) and Type-2 immune functions (Haim-Vilmovsky,et al., 2019) may provide additional therapeutic opportunities for diseases such as asthma, chronic obstructive pulmonary disease (COPD), and idiopathic pulmonary fibrosis (Gieseck et al., 2018).

However, like other ROR family members, RORα regulates multiple non-immune cell types, in non-inflammatory contexts. For example, *staggerer* mice, which carry a spontaneous deletion in *Rora*, have an underdeveloped cerebellar cortex, with deficiency in granule and Purkinje cells (Gold et al., 2007). RORα has also been linked to neurologic disorders, including autism, in humans (Devanna and Vernes, 2014; Nguyen et al., 2010; Sarachana and Hu, 2013). Significant circadian disruption, described in autistic patients (Hu et al., 2009; Melke et al., 2008; Nicholas et al., 2007), may be related to the role of RORα in regulation of the circadian clock (Jetten, 2009; Kojetin and Burris, 2014). Therefore, a deeper understanding of cell type-specific and context-dependent regulation of RORα is likely needed to inform strategies to combat RORα-associated immune diseases.

In summary, our study has elucidated a non-redundant role of RORα in Th17 lineage maintenance via reinforcement of the RORγt transcriptional program. Further characterization of the interaction of these two nuclear receptors may enable more refined strategies to target specific processes that fuel chronic inflammatory disease.

### Limitations of the study

Either due to limitations in methodology or antibody quality, the resolution of ChIP-seq experiments was insufficient to identify unique binding sites for RORα vs RORγt, if they exist, and to pinpoint the precise binding mode of these TFs, e.g. if there is interdependence for binding to distinct sites. The addition of corroborating ChIP-seq experiments in Th17 cells isolated from lymph nodes and tissues would further strengthen the conclusions based on *in vitro* differentiated cells. These points could be addressed in future studies using more sensitive ChIP-seq methods or chromatin binding assays, such as ChIP-exo or CUT&Tag, respectively. Sample size for the SC T_AKO_ Th17 RNA-seq condition was sub-optimal (n=2) as one sample was excluded from analysis due to presumed contamination (with reads in the deleted region of the *Rora* locus; all other T_AKO_ samples were devoid of reads in this region).

## STAR METHODS

### RESOURCE AVAILABILITY

#### Lead contact

Further information and requests for resources and reagents should be directed to and will be fulfilled by the Lead Contact, Dan R. Littman (Dan.Littman@med.nyu.edu).

#### Materials availability

- Mouse lines generated in this study have been deposited to the Jackson Laboratory.
- Accession numbers are listed in the key resources table.

#### Data and code availability

- The RNA-Seq, ATAC-Seq, ChIP-Seq datasets generated during this study have been deposited at Gene Expression Omnibus and are publicly available as of the date of publication. Accession numbers are listed in the key resources table.
- This paper does not report original code.
- Any additional information required to reanalyze the data reported in this paper is available from the lead contact upon request.

### EXPERIMENTAL MODEL AND SUBJECT DETAILS

#### Mouse Strains

All transgenic animals were bred and maintained in specific-pathogen free (SPF) conditions within the animal facility of the Skirball Institute at NYU School of Medicine. C57BL/6J mice were purchased from The Jackson Laboratory. Frozen sperm of *Rora* “knockout-first” mice (B6J.129S2-Rora^tm1.1Ics^/Ics) mice were obtained from the EMMA mouse repository and rederived onto a C57BL6/J background by NYU School of Medicine’s Rodent Genetic Engineering Core. Wildtype (WT), homozygous Rora floxed (*Rora*^fl/fl^) mice were generated by crossing animals with Tg(Pgk1-flpo)10Sykr mice purchased from The Jackson Laboratories. The flp3 transgene was removed before further breeding to with *CD4*^Cre^ (Tg(Cd4-^cre^)1Cwi/BfluJ). *Il17a*^eGFP^ reporter (JAX; C57BL/6-Il17a^tm1Bcgen^/J) mice were purchased from The Jackson Laboratories, and bred to the *Rorc* (JAX; B6(Cg)-Rorc^tm3Litt^/J) or *Rora* floxed mutant strains to generate the T_GKO_ (*CD4*^Cre^*Rorc(t)*^fl/fl^) or T_AKO_ (*CD4*^Cre^*Rora*^fl/fl^) strains, respectively. T_GKO_ or T_AKO_ strains were further bred to the CD45.1/1 (B6.SJL-Ptprca Pepcb/BoyJ) strain to generated congenically marked lines for co-transfer experiments and mixed bone marrow chimera generation. MOG-specific TCR transgenic (2D2, JAX; C57BL/6-Tg (Tcra2D2,Tcrb2D2)1 Kuch/J) mice were purchased from The Jackson Laboratories, maintained on CD45.1 background, and bred to the T_AKO_ strain. RAG1 knock-out (B6.129S7-Rag1tm1Mom/J) mice were purchased from The Jackson Laboratories, and maintained on CD45.1 background. SFB-specific TCR transgenic (7B8, JAX; C57BL/6-Tg(Tcra,Tcrb)2Litt/J) mice (Yang et al., 2014) were previously described, maintained on an Ly5.1 background, and bred to the T_AKO_ strain. RORA-TS mice were generated using CRISPR-Cas9 technology. Twin-Strep (TS) tag sequence was inserted into the last exon of the *Rora* locus in WT zygotes. Guide RNA and HDR donor template sequences are listed in Table S1. RORA-TS mice were bred with T_GKO_ mice to generate *Rorc* knock-out RORA-TS mice. RorgtTg (Rorgt-Cherry-CreERT2) and RorgtΔ+11kbTg (Rorgt-Cherry-CreERT2Δ+11kb) transgenic reporter mouse lines were generated by random insertion of bacterial artificial chromosomes (BACs) as described below. All in-house developed strains were generated by the Rodent Genetic Engineering Core (RGEC) at NYULMC. Age-(6–12 weeks) and sex-(both males and females) matched littermates stably colonized with Segmented Filamentous Bacteria (SFB) were used for all experiments. Before mating, the parental mice were orally gavaged with 1/4 pellet from SFB mono-associated mice to ensure stable colonization as described (Sano et al., 2015). To assay SFB colonization, SFB-specific 16S primers were used and universal 16S and/or host genomic DNA were quantified simultaneously to normalize SFB colonization in each sample. All animal procedures were performed in accordance with protocols approved by the Institutional Animal Care and Usage Committee of New York University School of Medicine and Yonsei University College of Medicine.

#### Generation of BAC transgenic reporter mice

BAC clone RP24–209K20 was obtained from CHORI (BAC PAC) and BAC DNA was prepared using the BAC100 kit (Clontech). Purified BAC DNA was then electroporated into the recombineering bacterial line SW105. The cassette containing 50bp homology arms surrounding the *Rorc(t)* translational start site ATG was linked to the mCherry-P2A-iCreERT2-FRT-Neo-FRT cassette by cloning into the pL451 vector. The resulting fragment was then excised using restriction digest and gel purified. Homologous recombination was performed by growing the BAC-containing SW105 cells to OD 600 and then heat shocking at 42°C for 15 minutes to induce expression of recombination machinery followed by cooling and washing with H_2_0 to generate electrocompetent cells. These were then electroporated with 0.1μg of purified targeting construct DNA. Correctly recombined bacteria were selected using chloramphenicol (BAC) and Kanamycin. The resultant BAC was purified, screened for integrity of BAC and recombineering junctions by PCR. This BAC was used subsequently to make scarless deletions of putative *cis*-regulatory elements using GalK positive negative selection according to the Soren Warming protocol #3. The primers, listed in Table S2, were used for generating amplicons for GalK recombineering, and screening for correct insertion and later removal of the GalK cassette.

The primers, listed in Table S3, were used for the recombineering that led to scarless deletion of *cis*-elements. Correct deletions were confirmed by PCR. The Neo cassette was removed in bacteria via Arabinose inducible Flipase expression and confirmed by PCR. To generate mice, purified BAC DNA was linearized by PI-SceI digestions, dialyzed using Injection buffer (10mM Tris-HCL pH 7.5, 0.1mM EDTA, 100mM NaCl, 30μM spermine, 70μM spermidine) to a concentration of 4ng/μl for microinjection into zygotes.

### METHOD DETAILS

#### *In vitro* T cell culture and phenotypic analysis

Mouse T cells were purified from lymph nodes and spleens of six to eight week old mice, by sorting live (DAPI-), CD4+CD25-CD62L+CD44low naïve T cells using a FACSAria (BD). Detailed antibody information is provided in the Key Resource Table. Cells were cultured in IMDM (Sigma) supplemented with 10% heat-inactivated FBS (Hyclone), 10U/ml penicillin-streptomycin (Invitrogen), 10μg/ml gentamicin (Gibco), 4mM L-glutamine, and 50μM β-mercaptoethanol. For T cell polarization, 1 × 105 cells were seeded in 200μl/well in 96-well plates that were pre-coated with a 1:20 dilution of goat anti-hamster IgG in PBS (STOCK = 1mg/ml, MP Biomedicals Catalog # 55398). Naïve T cells were primed with anti-CD3ε (0.25μg/mL) and anti-CD28 (1μg/mL) for 24 hours prior to polarization. Cells were further cultured for 48h under Th-lineage polarizing conditions; Th0 (Con. : 100U/mL IL-2, 2.5μg/mL anti-IL-4, 2.5μg/mL anti-IFN*γ*), Th17 (0.3 ng/mL TGF-β, 20 ng/mL IL-6, 20 ng/mL IL-23, 2.5μg/mL anti-IL-4, 2.5μg/mL anti-IFN*γ*).

#### Flow cytometry

Single cell suspensions were pelleted and resuspended with surface-staining antibodies in HEPES Buffered HBSS containing anti-CD16/anti-CD32. Staining was performed for 20–30min on ice. Surface-stained cells were washed and resuspended in live/dead fixable blue (ThermoFisher) for 5 minutes prior to fixation. PE and APC-conjugated MHC class II (I-Ab) MOG_38–49_ tetramers (GWYRSPFSRVVH) were provided by the NIH tetramer core facility. PE and APC-conjugated MHC class II (I-Ab) LT_166–178_ tetramers (RYYRNLNIAPAED) were produced and kindly provided by Timothy Hand’s laboratory at University of Pittsburgh. Staining of tetramer positive T cells was carried out after magnetic isolation of the cells as described (Moon et al., 2009). All tetramer stains were performed at room temperature for 45–60 minutes. For transcription factor staining, cells were treated using the FoxP3 staining buffer set from eBioscience according to the manufacturer’s protocol. Intracellular stains were prepared in 1X eBioscience permwash buffer containing normal mouse IgG (conc), and normal rat IgG (conc). Staining was performed for 30–60min on ice. For cytokine analysis, cells were initially incubated for 3h in RPMI or IMDM with 10% FBS, phorbol 12-myristate 13-acetate (PMA) (50 ng/ml; Sigma), ionomycin (500 ng/ml;Sigma) and GolgiStop (BD). After surface and live/dead staining, cells were treated using the Cytofix/Cytoperm buffer set from BD Biosciences according to the manufacturer’s protocol. Intracellular stains were prepared in BD permwash in the same manner used for transcription factor staining. For EdU staining, we followed manufacturer’s instruction (EdU Flow Cytometry Kit, baseclick). Absolute numbers of isolated cells from peripheral mouse tissues in all studies were determined by comparing the ratio of cell events to bead events of CountBright^™^ absolute counting beads. Flow cytometric analysis was performed on an LSR II (BD Biosciences) or an Aria II (BD Biosciences) and analyzed using FlowJo software (Tree Star).

#### Induction of EAE by MOG-immunization

For induction of active experimental autoimmune encephalomyelitis (EAE), mice were immunized subcutaneously on day 0 with 100μg of MOG_35–55_ peptide, emulsified in CFA (Complete Freund’s Adjuvant supplemented with 2mg/mL Mycobacterium tuberculosis H37Ra), and injected i.p. on days 0 and 2 with 200 ng pertussis toxin (Calbiochem). For 2D2 transfer EAE experiments, after retrovirus transduction and/or CAS9/RNP electroporation (described below), CD45.1/2 T_WT_ and CD45.2/2 T_AKO_ 2D2 cells were differentiated to ROR*γ*t+ effector Th17 cells under the Th17 polarizing condition in vitro for 4 days, then were mixed 1:1 and injected intravenously into recipient mice at total 2 × 105 ROR*γ*t+ 2D2 cells per recipient (*CD4*^Cre^/CD45.1/1). The recipient mice were subsequently immunized for inducing EAE. The EAE scoring system was as follows: 0-no disease, 1- Partially limp tail; 2- Paralyzed tail; 3- Hind limb paresis, uncoordinated movement; 4- One hind limb paralyzed; 5- Both hind limbs paralyzed; 6- Hind limbs paralyzed, weakness in forelimbs; 7- Hind limbs paralyzed, one forelimb paralyzed; 8- Hind limbs paralyzed, both forelimbs paralyzed; 9- Moribund; 10- Death. For isolating mononuclear cells from spinal cords during EAE, spinal cords were mechanically disrupted and dissociated in RPMI containing collagenase (1 mg/ml collagenaseD; Roche), DNase I (100 μg/ml; Sigma) and 10% FBS at 37 °C for 30 min. Leukocytes were collected at the interface of a 40%/80% Percoll gradient (GE Healthcare).

#### Retroviral reconstitution of *Rora* or the RORα -target genes into T_AKO_ 2D2 cells

To generate the ectopic expression retrovirus vector, mouse *Rora*, *Rorc(t)* and *Bhlhe40* were subcloned into the retroviral vector, MSCV-IRES-Thy1.1 (MiT). MiT-Rora, MiT-Rorc(t), MiT-Bhlhe40, and MiT (“empty” vector) plasmids were transfected into PLAT-E retroviral packaging cell line (Cell Bioloab, INC.) using TransIT^®^-293 transfection reagent (Mirus). Supernatants were collected at 48 h after transfection. Naive T_WT_ or T_AKO_ 2D2 cells were isolated and activated by plate-bound anti-CD3 and anti-CD28. 24 hours after activation, cells were spin- infected by retroviruses MiT-Rora, MiT-Bhlhe40 or control empty vector (MiT-Empty) as described previously (Skon et al., 2013), then were further cultured for 96hrs under Th17-lineage polarizing condition; 20 ng/mL IL-6, 20 ng/mL IL-23, 2.5μg/mL anti-IL-4, 2.5μg/mL anti- IFN*γ*. Prior to adoptive transfer into recipients, Thy1.1^+^ transduced cells were labeled and enriched with EasySep^™^ Mouse CD90.1 Positive Selection Kit (STEMCELL).

#### CRISPR mutation of RORE in the +11kb *cis*-element of *Rorc* in 2D2 T cells

To mutate RORE in the +11kb *cis*-regulatory element of *Rorc*, we delivered CRISPR-Cas9 ribonucleoprotein (RNP) complexes, containing Alt-R CRISPR-Cas9 guide RNAs (the RORE targeting or control sgRNA sequences are listed in the table of STAR Methods) and Cas9 nuclease, into 2D2 cells using electroporation with the Amaxa Nucleofector system (Lonza); 20 μM (1:1.2, Cas9:sgRNA) Alt-R (Integrated DNA Technologies, Inc) Cas9 RNP complex, and 20 μM Alt-R Cas9 Electroporation Enhancer (Integrated DNA Technologies, Inc) as described previously (Vakulskas et al., 2018). sgRNAs were designed using the Crispr guide design software (Integrated DNA Technologies, Inc). FACS-sorted naïve (CD4+CD8-CD25- CD62L+CD44low) 2D2 T cells were primed for 18 hrs in T cell medium (RPMI supplemented with 10% FCS, 2mM b-mercaptoethanol, 2mM glutamine), along with anti-CD3 (BioXcell, clone 145–2C11, 0.25 mg/ml) and anti-CD28 (BioXcell, clone 37.5.1, 1 mg/ml) antibodies on tissue culture plates, coated with polyclonal goat anti-hamster IgG (MP Biomedicals). RNPs were formed by the addition of purified Cas9 protein to sgRNAs in 1 × PBS. Complexes were allowed to form for 30 min at 37°C before electroporation. RNP complexes (5 μL) and 1×106 2D2 cells (20 μL) were mixed and electroporated according to the manufacturer’s specifications using protocol DN-100 (P3 Primary Cell 4D-NucleofectorTM). After 4hrs of recovery in pre-warmed T cell culture medium (Mouse T Cell NucleofectorTM Medium), the electroporated 2D2 cells were polarized into Th17 cells for 96hrs under Th17-lineage polarizing condition; 20 ng/mL IL-6, 20 ng/mL IL- 23, 2.5μg/mL anti-IL-4, 2.5μg/mL anti-IFN*γ*. For *Rora* reconstitution experiment described in Figure S6C, MiT-Rora, MiT-Rorc(t) and MiT (empty) retrovirus were transduced after 24hrs of the electroporation. Prior to adoptive transfer into recipients, Thy1.1+ transduced cells were labeled and enriched with EasySep^™^ Mouse CD90.1 Positive Selection Kit (STEMCELL). The genome targeting efficiency was determined by T7 endonuclease assay (NEB) followed by manufacturer’s protocol (Figure S6A). In parallel, RORE locus of the +11kb *cis*-element of *Rorc(t)* locus was PCR amplified and cloned into pCR^™^2.1 vector (ThermoFisher), and mutations in the RORE locus was confirmed by sanger sequencing of the clones (Figure S6B).

#### Generation of bone marrow (BM) chimeric reconstituted mice

Bone marrow (BM) mononuclear cells were isolated from donor mice by flushing the long bones. To generate T_WT_/T_GKO_ chimeric reconstituted mice, CD45.1/2 T_WT_ (*CD4*^Cre^*Rorc*^+/+^) and CD45.2/2 T_GKO_ (CD4^Cre^Rorc^fl/fl^) mice were used as donors. To generate T_WT_/T_AKO_ chimeric reconstituted mice, CD45.1/2 T_WT_ (*CD4*^Cre^*Rora*^+/+^) and CD45.2/2 T_AKO_ (*CD4*^Cre^*Rora*^fl/f^*l*) mice were used as donors. Red blood cells were lysed with ACK Lysing Buffer, and lymphocytes were labeled with Thy1.2 magnetic microbeads and depleted with a Miltenyi LD column. The remaining cells were resuspended in PBS for injection in at 1:4 (T_WT_:T_GKO_) or 1:1 ratio (T_WT_: T_AKO_) to achieve 1:1 chimerism of peripheral T cell populations. Total 5×10^6^ mixed BM cells were injected intravenously into 6 week old RAG1 knock-out recipient mice that were irradiated 4h before reconstitution using 1000 rads/mouse (2×500rads, at an interval of 3h, at X-RAD 320 X-Ray Irradiator). Peripheral blood samples were collected and analyzed by FACS 7 weeks later to check for reconstitution.

#### Oral vaccination

Double mutant E. coli heat labile toxin (R192G/L211A) (dmLT), was produced from *E. coli* clones expressing recombinant protein as previously described (Norton et al., 2011). Mice were immunized twice, 7 days apart by oral gavage, and vaccine responses were assayed 2 weeks after primary gavage as described before (Hall et al., 2008).

#### Isolation of lamina propria lymphocytes

The intestine (small and/or large) was removed immediately after euthanasia, carefully stripped of mesenteric fat and Peyer’s patches/cecal patch, sliced longitudinally and vigorously washed in cold HEPES buffered (25mM), divalent cation-free HBSS to remove all fecal traces. The tissue was cut into 1-inch fragments and placed in a 50ml conical containing 10ml of HEPES buffered (25mM), divalent cation-free HBSS and 1 mM of fresh DTT. The conical was placed in a bacterial shaker set to 37 °C and 200rpm for 10 minutes. After 45 seconds of vigorously shaking the conical by hand, the tissue was moved to a fresh conical containing 10ml of HEPES buffered (25mM), divalent cation-free HBSS and 5 mM of EDTA. The conical was placed in a bacterial shaker set to 37 °C and 200rpm for 10 minutes. After 45 seconds of vigorously shaking the conical by hand, the EDTA wash was repeated once more in order to completely remove epithelial cells. The tissue was minced and digested in 5–7ml of 10% FBS-supplemented RPMI containing collagenase (1 mg/ml collagenaseD; Roche), DNase I (100 μg/ml; Sigma), dispase (0.05 U/ml; Worthington) and subjected to constant shaking at 155rpm, 37 °C for 35 min (small intestine) or 55 min (large intestine). Digested tissue was vigorously shaken by hand for 2 min before adding 2 volumes of media and subsequently passed through a 70 μm cell strainer. The tissue was spun down and resuspended in 40% buffered percoll solution, which was then aliquoted into a 15ml conical. An equal volume of 80% buffered percoll solution was underlaid to create a sharp interface. The tube was spun at 2200rpm for 22 minutes at 22 °C to enrich for live mononuclear cells. Lamina propria (LP) lymphocytes were collected from the interface and washed once prior to staining.

#### SFB-specific T cell proliferation assay

Sorted naive 7B8 or 2D2 CD45.1/1 CD4 T cells were stained with CellTrace^™^ Violet Cell Proliferation Kit (Life Technology) followed by manufacturer’s protocol. Labeled cells were administered into SFB-colonized congenic CD45.2/2 recipient mouse by i.v. injection. MLNs of the SFB-colonized mice were collected at 96h post transfer for cell division analysis.

#### RNA isolation and library preparation for RNA sequencing

Total RNAs from in vitro polarized T cells or sorted cell populations were extracted using TRIzol (Invitrogen) followed by DNase I (Qiagen) treatment and cleanup with RNeasy MinElute kit (Qiagen) following manufacturer protocols. RNA-Seq libraries for *ex vivo* isolated IL17^eGFP+^ T_WT_ or T_AKO_ Th17 lineages from DLN or spinal cords of immunized BM chimeras at peak of EAE were prepared with the SMART-Seq^®^ v4 PLUS Kit (Takara, R400752). The sequencing was performed using the Illumina NovaSeq or NextSeq. RNA-seq libraries were prepared and sequenced by the Genome Technology Core at New York University School of Medicine.

#### Library preparation for ATAC sequencing

Samples were prepared as previously described (Buenrostro et al., 2013). Briefly, 50,000 sort-purified Th17 cells were pelleted in a fixed rotor centrifuge at 500xg for 5 minutes, washed once with 50 μL of cold 1x PBS buffer. Spun down again at 500xg for 5 min. Cells were gently pipetted to resuspend the cell pellet in 50 μL of cold lysis buffer (10 mM Tris-HCl, pH7.4, 10 mM NaCl, 3 mM MgCl2, 0.1% IGEPAL CA-630) for 10 minutes. Cells were then spun down immediately at 500xg for 10 min and 4 degrees after which the supernatant was discarded and proceeded immediately to the Tn5 transposition reaction. Gently pipette to resuspend nuclei in the transposition reaction mix. Incubate the transposition reaction at 37 degrees for 30 min. Immediately following transposition, purify using a Qiagen MinElute Kit. Elute transposed DNA in 10 μL Elution Buffer (10mM Tris buffer, pH 8). Purified DNA can be stored at ^™^20 degrees C. The transposed nuclei were then amplified using NEBNext High-fidelity 2X PCR master mix for 5 cycles. In order to reduce GC and size bias in PCR, the PCR reaction is monitored using qPCR to stop amplification prior to saturation using a qPCR side reaction. The additional number of cycles needed for the remaining 45 μL PCR reaction is determined as following: (1) Plot linear Rn vs. Cycle (2) Set 5000 RF threshold (3) Calculate the # of cycle that is corresponded to ¼ of maximum fluorescent intensity. Purify amplified library using Qiagen PCR Cleanup Kit. Elute the purified library in 20 μL Elution Buffer (10mM Tris Buffer, pH 8). Be sure to dry the column before adding elution buffer. The purified libraries were then run on a high sensitivity Tapestation to determine if proper tagmentation was achieved (band pattern, not too much large untagmented DNA or small overtagmented DNA at the top or bottom of gel. Paired-end 50bp sequences were generated from samples on an Illumina HiSeq2500.

#### Library preparation for Chromatin Immunoprecipitation for sequencing (ChIP-Seq)

RORα-TS and RORγt ChIP-Seq was performed as described (Ciofani et al., 2012) with the following modifications. For each ChIP, 20–80 million cells were cross-linked with paraformaldehyde; chromatin was isolated using truChIP Chromatin Shearing Kit (Covaris) and fragmented with a S220 Focused-ultrasonicator (Covaris). Twin-strep (TS) tagged RORα protein was precipitated using Strep-TactinXT according to the manufacturer’s protocol (IBA Lifesciences). Following immunoprecipitation, the protein-DNA crosslinks were reversed and DNA was purified. DNA from control samples was prepared similarly but without immunoprecipitation. Sequencing libraries were made from the resulting DNA fragments for both ChIP and controls using DNA SMART^™^ ChIP-Seq Kit (Takara) for RORα-TS ChIP-Seq and KAPA HyperPlus Kit (Roche) for RORγt ChIP-Seq. The ChIP-Seq libraries were sequenced with paired-end 50 bp reads on an Illumina HiSeq 4000.

#### Chromatin Immunoprecipitation for quantitative PCR analysis (ChIP-qPCR)

To generate the ectopic expression of T-bet in committed Th17 cells, mouse *Tbx21* was subcloned into the retroviral vector, MSCV-IRES-Thy1.1 (MiT). As described above, plasmids encoding control or *Tbx21* were transfected into PLAT-E retroviral packaging cells (Cell Biolabs, Inc.) using TransIT^®^^™^293 transfection reagent (Mirus). Supernatants were collected at 48h after transfection. After 48h Th17 polarization (20 ng/mL IL-6, 0.1ng/ml TGF-β, 20 ng/mL IL-23, 2.5μg/mL anti-IL-4, 2.5μg/mL anti-IFNγ), cells were spin-infected by retroviruses MiT-Tbx21 or control empty vector (MiT-Empty) as described above, then were further cultured for 48h under the Th17-lineage polarizing condition. Prior to ChIP analysis, Thy1.1+ transduced cells were labeled and enriched with EasySep^™^ Mouse CD90.1 Positive Selection Kit (STEMCELL). For ChIP analysis, rabbit anti-RORα and isotype control antibodies were used to precipitate targeted DNA fragments (#9005, Cell Signaling Technology). The anti-RORα rabbit polyclonal antibody was generated against amino acids 121–267 of RORα, and affinity purified antibody was isolated from serum using recombinant RORα protein. For each ChIP, 10 million cells were cross-linked with paraformaldehyde, then the chromatin was isolated using a Bioruptor (Diagenode). Enrichment of genomic DNA fragments by ChIP was validated by Realtime PCR (QuantStudio 5 Real-Time PCR Instrument, Applied Biosystems) with primers (KEY RESOURCE TABLE) targeting the *Il23r* and *Il17a* promoter regions, as well as the *Rorc(t)* +11 kb *cis*-regulatory element locus. Primers targeting the *Actb* promotor region functioned as the control for the ChIP assay.

### QUANTIFICATION AND STATISTICAL ANALYSIS

#### Transcriptome analysis

##### RNA-Seq methods:

Bulk RNA-Seq fastq files were aligned to the mm10 reference genome using star v 2.7.3a. Bam files were converted to bigwig files via deeptools v 3.3.0 bamCoverage for visualization. DEseq2 was used for differential gene analysis.

##### ChIP-Seq methods:

ChIP-Seq fastq files were aligned to the mm10 reference genome using star v 2.7.3a. Bam files were converted to bigwig files via deeptools v 3.3.0 bamCoverage and normalized by RPGC to compare peak heights across samples. Deeptools computeMatrix and plotHeatmap were used to make heatmaps. Macs2 was used to call peaks using a significance cutoff of 0.01 for the previously published RORγt ChIP-Seq dataset (Ciofani et al., 2012) (Figure S4E, left panel), 0.5 for the RORα-Twin Strep ChIP-Seq datasets (Figure S4E, middle and right panels), and 0.05 for the RORγt ChIP-Seq datasets (Figure S4I). During peak calling the treatment file was used with its associated control file. The homer annotatePeaks.pl script was used to annotate peaks within 10kb of a gene.

##### ATAC-Seq methods:

Bowtie2 was used to align the reads to the mm10 genome using parameters - very-sensitive. Picard tools was used to mark and remove duplicates. Deeptools bamCoverage was used to generate a bigwig file normalized using RPGC.

#### Statistical analysis

Differences between groups were calculated using the unpaired two-sided Welch’s t-test or the two-stage step-up method of Benjamini, Krieger and Yekutieliun. For EAE disease induction, log-rank test using the Mantal-Cox method was performed. For RNA-seq analysis, differentially expressed genes were calculated in DESeq2 using the Wald test with Benjamini–Hochberg correction to determine the FDR. Genes were considered differentially expressed with FDR < 0.01 and log2 fold change > 1.2. Data was processed with GraphPad Prism, Version 8 (GraphPad Software). We treated less than 0.05 of p value as significant differences. *p < 0.05, **p < 0.01, ***p < 0.001, and ****p < 0.0001. Details regarding number of replicates and the definition of center/error bars can be found in figure legends.

## Supplementary Material

1

## Figures and Tables

**Table:** KEY RESOURCE TABLE

REAGENT or RESOURCE	SOURCE	IDENTIFIER
Antibodies		
Flow Cytometry: anti-mouse CD3 (17A2) AlexaFluor700	ThermoFisher	Cat. 56–0032
Flow Cytometry: anti-mouse CD4 (RM4–5) eFluor450	ThermoFisher	Cat. 48–0042
Flow Cytometry: anti-mouse CD11b (M1/70) PerCP-cy5.5	ThermoFisher	Cat. 45–0112
Flow Cytometry: anti-mouse CD11c (N418) PerCP-cy5.5	ThermoFisher	Cat. 45–0114
Flow Cytometry: anti-mouse CD14 (Sa2–8) FITC	ThermoFisher	Cat. 11–0141
Flow Cytometry: anti-mouse CD14 (Sa2–8) PerCP-cy5.5	ThermoFisher	Cat. 45–0141
Flow Cytometry: anti-mouse CD19 (1D3) PerCP-cy5.5	TONBO	Cat. 65–0193
Flow Cytometry: anti-mouse CD25 (PC61) PE-Cy7	TONBO	Cat. 60–0251
Flow Cytometry: anti-mouse CD44 (IM7) BV500	BD Bioscience	Cat. 563114
Flow Cytometry: anti-mouse CD45.1 (A20) BV650	BD Bioscience	Cat. 563754
Flow Cytometry: anti-mouse CD45.2 (104) APC-e780	ThermoFisher	Cat. 47–0454
Flow Cytometry: anti-mouse CD62L (MEL-14) APC	ThermoFisher	Cat. A14720
Flow Cytometry: anti-mouse TCRβ (H57–597) PerCP-cy5.5	ThermoFisher	Cat. 45–5961
Flow Cytometry: anti-mouse TCRβ (H57–597) BV711	BD Bioscience	Cat. 563135
Flow Cytometry: anti-mouse TCR Vβ3.2 (RR3–16) FITC	ThermoFisher	Cat. 11–5799
Flow Cytometry: anti-mouse TCR Vβ6 (RR4–7) FITC	BD Bioscience	Cat. 553193
Flow Cytometry: anti-mouse MHCII (M5/114.15.2) PE	ThermoFisher	Cat. 12–5321
Flow Cytometry: anti-mouse MHCII (M5/114.15.2) PerCP-cy5.5	BD Bioscience	Cat. 562363
Flow Cytometry: anti-mouse FoxP3 (FJK-16s) FITC	ThermoFisher	Cat. 53–5773
Flow Cytometry: anti-mouse RORγt (B2D) PE	ThermoFisher	Cat. 12–6981
Flow Cytometry: anti-mouse RORγt (Q31–378) BV421	BD Bioscience	Cat. 562894
Flow Cytometry: anti-mouse T-bet (eBio4B10) PE-cy7	ThermoFisher	Cat. 25–5825
Flow Cytometry: anti-mouse IL-17A (eBio17B7) eFluor660	ThermoFisher	Cat. 50–7177
Flow Cytometry: anti-mouse IL-17F (9D3.1C8) AlexaFluor488	Biolegend	Cat. 517006
Flow Cytometry: anti-mouse IFNγ (XM61.2) eFluor450	ThermoFisher	Cat. 48–7311
*In vitro* T cell differentiation: anti-hamster IgGs	MP Biomedicals Catalog	Cat. 55398
*In vitro* T cell differentiation: anti-mouse CD3ε (145–2C11)	BioXCell	Cat. BP0001–1
*In vitro* T cell differentiation: anti-mouse CD28 (37.51)	BioXCell	Cat. BE0015–1
*In vitro* T cell differentiation: anti-mouse IL-4 (11B11)	BioXCell	Cat. BP0045
*In vitro* T cell differentiation: anti-mouse IFNγ (XMG1.2)	BioXCell	Cat. BP0055
RORα ChIP qPCR : polyclonal rabbit anti-mouse RORα	This paper	N/A
		
Biological Samples		
Fetal Bovine Serum	Atlanta Biologicals	Cat. S11195 Lot. A16003
		
Chemicals, Peptides, and Recombinant Proteins		
EDTA, 0.5M, pH8.0	Ambion	Cat. AM9260G
TransIT^®^-293 Transfection Reagent	Mirus	Cat. MIR2704
Collagenase D	Roche	Cat. 11088882001
Dispase	Worthington	Cat. LS02104
DNase I	Sigma	Cat. DN25
DTT	Sigma	Cat. D9779
Percoll	GE Healthcare Life Sciences	Cat. 45001747
Ficoll-Paque Premium	GE Healthcare Life Sciences	Cat. 17–5442-02
2-Mercaptoethanol (BME)	ThermoFisher	Cat. 21985023
Phorbol Myristate Acetate	Sigma	Cat. P1585
Ionomycin	Sigma	Cat. I0634
Recombinant Human IL-2	NIH AIDS Reagent Program	Cat. 136
Recombinant Human TGFβ Protein	Peprotech	Cat. 100–21-10ug
Recombinant Mouse IL-6 Protein	R&D systems	Cat. 406-ML-200/CF
Recombinant Mouse IL-23 Protein	R&D systems	Cat. 1887-ML
Alt-R^®^ S.p. HiFi Cas9 Nuclease V3	Integrated DNA Technologies	Lot #0000473804, 0000469029
Alt-R^®^ Cas9 Electroporation Enhancer	Integrated DNA Technologies	Lot #0000472336
		
Critical Commercial Assays		
LIVE/DEAD^®^ Fixable Blue Dead Cell Stain Kit	ThermoFisher	Cat. L34961
CountBright^™^ absolute counting beads	ThermoFisher	Cat. C36950
BD Cytofix/Cytoperm Plus Fixation/Permeabilization Solution Kit	BD Biosciences	Cat. 554714
eBioscience^™^ Foxp3 / Transcription Factor Staining Buffer Set	ThermoFisher	Cat. 00–5523-00
LightCycler^®^ 480 SYBR Green I Master	Roche Life Science	Cat. 04707516001
SuperScript^™^ III First-Strand Synthesis System	ThermoFisher	Cat. 18080051
RNeasy Mini Kit	QIAGEN	Cat. 74104
RNeasy MinElute Cleanup Kit	QIAGEN	Cat. 74204
RNase-Free DNase Set	QIAGEN	Cat. 79254
TRIzol^™^ Reagent	ThermoFisher	Cat. 15596026
BD GolgiPlug Protein Transport Inhibitor	BD Biosciences	Cat. 555029
BD GolgiStop Protein Transport Inhibitor	BD Biosciences	Cat. 554724
EdU Flow Cytometry 647–50 Kit + EdU	Baseclick	Cat. BCK647-IV-FC -M
CellTrace^™^ Violet Cell Proliferation Kit, for flow cytometry	ThermoFisher	Cat. C34557
EasySep^™^ Mouse CD90.1 Positive Selection Kit	STEMCELL	Cat. 18958
T7 Endonuclease I	NEB	Cat. M0302
TA Cloning Kits	ThermoFisher	Cat. K202020
DNA SMART^™^ ChIP-Seq Kit	Takara	Cat. 634865
KAPA HyperPlus Kit	Roche	Cat. 07962380001
Mouse T Cell NucleofectorTM Medium	Lonza	Cat. VZB-1001
P3 Primary Cell 4D-NucleofectorTM X Kit S	Lonza	Cat. V4XP-3032
truChIP Chromatin Shearing Kit with Formaldehyde	Covaris	Cat. 520154
SimpleChIP^®^ Enzymatic Chromatin IP Kit (Magnetic Beads)	Cell Signaling Thechnology	Cat. 9003
		
Deposited Data		
RNA-Seq raw and analyzed data : *ex vivo* RNA-Seq of sort-purified T_WT_ (CD4^Cre^) or T_AKO_ (CD4^Cre^Rora^fl/fl^ ) Th17 (IL17A^eGFP+^) cells from draining lymph nodes or spinal cords of the mixed bone marrow chimera mice at the peak of EAE disease	This paper	GEO: GSE163338
ATAC-Seq raw and analyzed data	This paper	GEO: GSE163340
: ATAC-Seq analysis of *in vitro* polarized or *ex vivo* sort-purified Th17 cells (IL17A^eGFP+^)		
ChIP-Seq raw and analyzed data : RORα-TwinStrep (TS) ChIP-Seq analysis of *in vitro* polarized T_WT_ (RORα-TS) or T_GKO_ (CD4^Cre^Rora^fl/fl^RORα-TS) Th17 cells	This paper	GEO: GSE163339
ChIP-Seq raw and analyzed data : RORγt ChIP-Seq analysis of *in vitro* polarized T_WT_ (CD4^Cre^) or T_AKO_ (CD4^Cre^Rora^fl/fl^) or T_DKO_ (CD4^Cre^Rora^fl/fl^Rorc^fl/fl^) Th17 cells	This paper	GEO: GSE163341
		
Experimental Models: Cell Lines		
Plat-E Retroviral Packaging Cell Line	Cell Biolabs, INC.	Cat. RV-101
		
Experimental Models: Organisms/Strains		
C57BL/6J	The Jackson Laboratory	JAX:000664
C57BL/6-Il17a^tm1Bcgen^/J	The Jackson Laboratory	JAX: 018472
B6. SJL Ptprc^a^ Pepc^b^/BoyJ	The Jackson Laboratory	JAX:002014
C57BL/6-Tg(Tcra2D2,Tcrb2D2)1Kuch/J	The Jackson Laboratory	JAX:006912
Tg(Cd4-^cre^)1Cwi/BfluJ	The Jackson Laboratory	JAX: 017336
C57BL/6-Tg(Tcra,Tcrb)2Litt/J	The Jackson Laboratory	JAX: 027230
B6.129S7-Rag1^tm1Mom^/J	The Jackson Laboratory	JAX: 002216
B6(Cg)-Rorc^tm3Litt^/J	The Jackson Laboratory	JAX: 008771
B6J.129S2-Rora^tm1.1Ics^/Ics	The EMMA mouse repository	EM:12934
RorgtTg(Rorgt-Cherry-CreERT2)	This paper	N/A
RorgtΔ+11kbTg(Rorgt-Cherry-CreERT2Δ+11kb)	This paper	N/A
B6.129P2(Cg)-Rorctm2Litt/J	The Jackson Laboratory	JAX: 007572
RORα -TwinStrep(TS)	This paper	JAX: 035700
		
Oligonucleotides		
MSCV-IRES-Thy1.1 DEST	Addgene	Plasmid #17442
Control (Olfr2) sgRNA mA*mC*mG*rArUrUrCrCrUrArArGrArUrGrCrUrUrG rCrGrUrUrUrUrArGrArGrCrUrArGrArArArUrArGrCr ArArGrUrUrArArArArUrArArGrGrCrUrArGrUrCrCrG rUrUrArUrCrArArCrUrUrGrArArArArArGrUrGrGrCr ArCrCrGrArGrUrCrGrGrUrGrCmU*mU*mU*rU	Integrated DNA Technologies	N/A
+11kb targeting sgRNA mU*mG*mG*rUrGrArGrUrArUrCrUrArGrGrUrCrAr CrCrGrUrUrUrUrArGrArGrCrUrArGrArArArUrArGr CrArArGrUrUrArArArArUrArArGrGrCrUrArGrUrCrC rGrUrUrArUrCrArArCrUrUrGrArArArArArGrUrGrGr CrArCrCrGrArGrUrCrGrGrUrGrCmU*mU*mU*rU	Integrated DNA Technologies	N/A
Rorc_11kb_T7assay forward primer GTTCTTCTACCCACAGCCCT	This Paper	N/A
Rorc_11kb_T7assay reverse primer CCATTTCCCCAGCTCTGTCT	This Paper	N/A
Forward primer for T7 endonuclease assay for determining genome targeting efficiency of +11kb *Rorc cis*-element: GTTCTTCTACCCACAGCCCT	This paper	N/A
Reverse primer for T7 endonuclease assay for determining genome targeting efficiency of +11kb *Rorc cis*-element: CCATTTCCCCAGCTCTGTCT	This paper	N/A
Primer sequences for qPCR analysis	Table S4	N/A
		
Software and Algorithms		
FlowJo	9.9.6	https://www.flowjo.com/
Prism	8.1.0	https://www.graphpad.com/scientific-software/prism/
IMARIS software	9.0.1	Oxford Instruments
DEseq2	1.22.2	https://bioconductor.org/packages/release/bioc/html/DESeq2.html
Gene Set Enrichment Analysis tool	3.0	http://software.broadinstitute.org/gsea/index.jsp
star	2.7.3a.	https://github.com/alexdobin/STAR
Macs2		https://github.com/macs3-project/MACS
deeptools	3.3.0	https://deeptools.readthedocs.io/en/develop/
IGV	2.3.91	http://software.broadinstitute.org/software/igv/
homer	4.10	http://homer.ucsd.edu/homer/

## Highlights

- RORα is required for sustained Th17 responses *in vivo*.
- RORα shares genomic binding sites with RORγt.
- RORα binding depends on RORγt while RORγt can bind ROREs in the absence of RORα.
- A *Rorc(t)* +11kb *cis*-element is required for RORα-maintained RORγt expression *in vivo.*
