# Supplementary material for: Transcription factor RORα enforces stability of the Th17 cell effector program by binding to a Rorc cis-regulatory element
Source: Immunity. Author manuscript; Available in PMC 2022 Dec 16. (PMC9757081; doi:10.1016/j.immuni.2022.09.013)
Supplement: 1 [file NIHMS1843752-supplement-1.pdf]

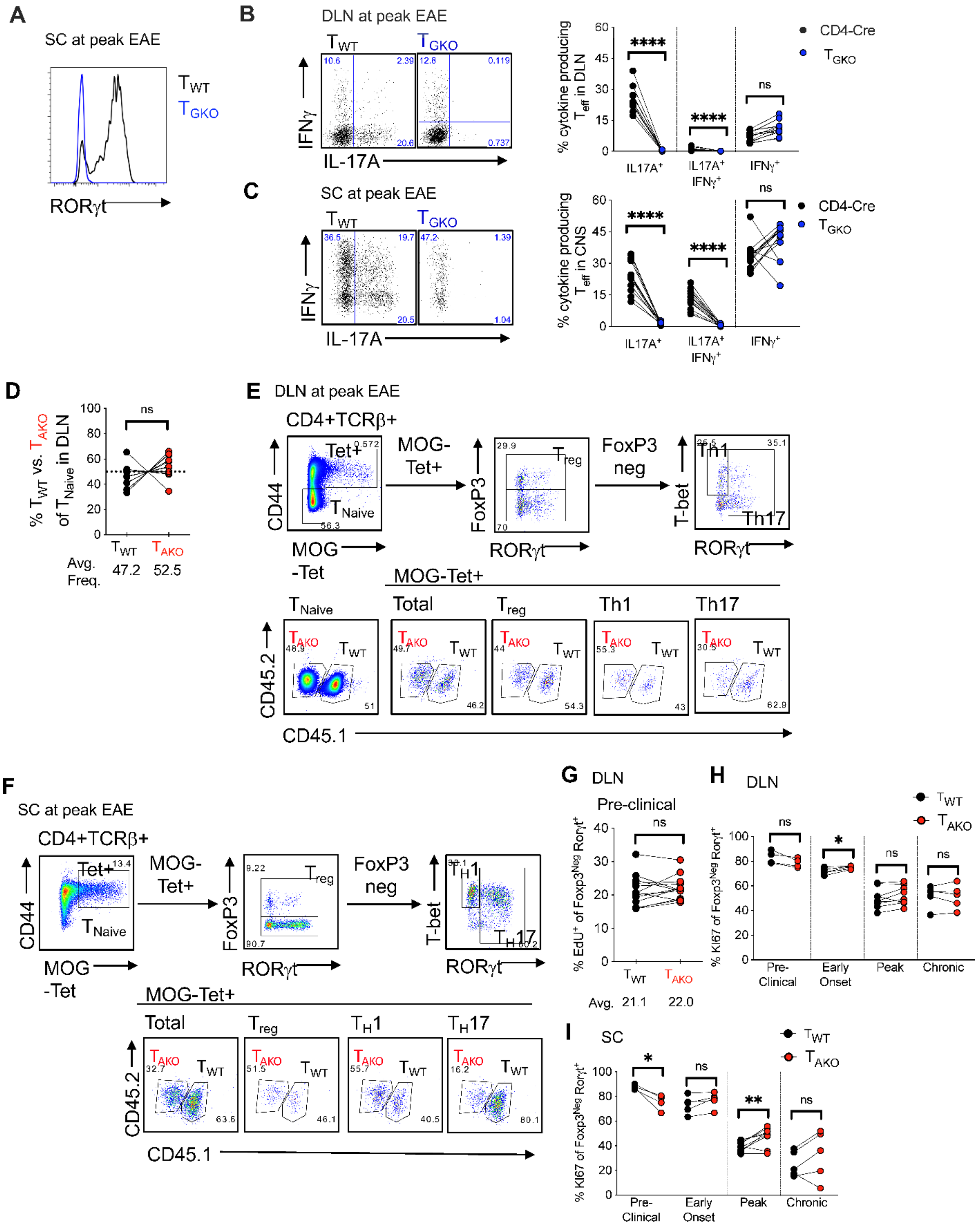

Figure S1: RORα and RORγt are differentially required for Th17 cell differentiation and accumulation during autoimmune encephalomyelitis. Related to Figure 1.

(A) Validation of *Rorc* inactivation in *ex vivo* isolated T_{WT} vs. T_{GKO} cells from a spinal cord of a mixed bone marrow chimera mouse at peak of EAE.

(B and C) IL-17A and IFNγ production of $CD44^{hi}$ effector T cells upon their *ex vivo* PMA/Iono restimulation. Cells from DLN (B) and SC (C) of T_{WT}/T_{GKO} BM chimera at peak of EAE. Data combined three experiments with 13 BM chimera mice.

(D) Mean percent donor-derived $CD44^{lo}$ $CD4^{+}$ naïve T cell chimerism at peak of EAE, as determined by flow cytometric analysis of DLN. Data combined three experiments with 12 T_{WT}/T_{AKO} BM chimera mice.

(E and F) Gating strategies to identify all Th populations amongst MOG-tetramer^{+} T_{WT} and T_{AKO} donor-derived $CD4^{+}$ T cells in the DLN (E) and SC (F) of T_{WT}/T_{AKO} BM chimera mice at peak of EAE.

(G) Percent of EdU-incorporating Th17 (RORγt^{+}FoxP3^{neg}) cells from DLN of T_{WT}/T_{AKO} BM chimera mice at pre-clinical stage of EAE. Data combined with 13 T_{WT}/T_{AKO} BM chimera mice.

(H and I) Percent of Ki-67^{+} Th17 (RORγt^{+}/FoxP3^{neg}) cells from DLN (H) and SC (I) of T_{WT}/T_{AKO} BM chimera mice at indicated stages of EAE. Data combined two experiments for the pre-clinical (n=4), early onset (n=5), acute (n=9), and chronic stages (n=5) of disease, respectively. Statistics were calculated using the paired sample T test. ns = not significant, *$p < 0.05$, **$p < 0.01$, ****$p < 0.0001$.

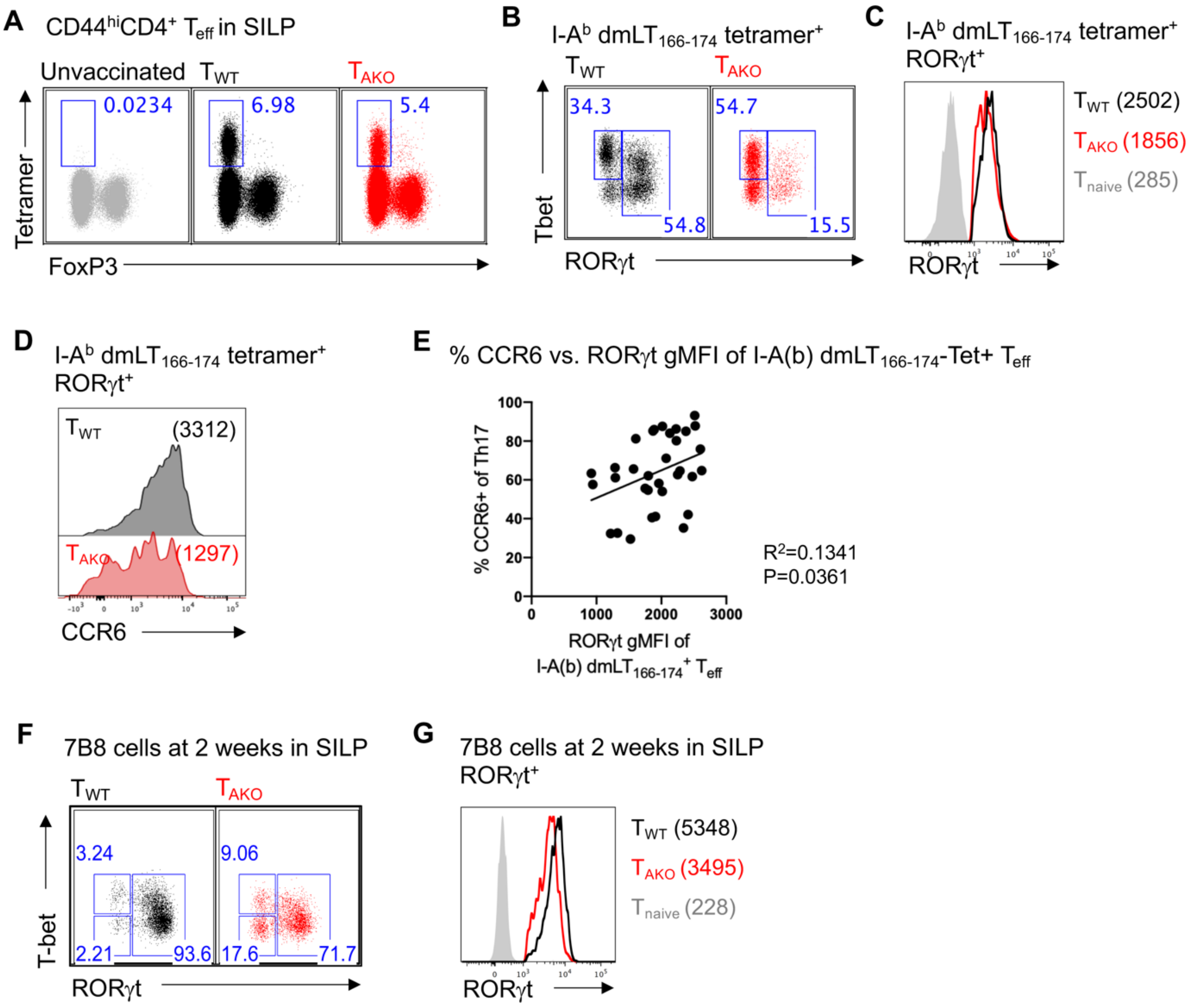

Figure S2. RORα deficiency impairs Th17 cell accumulation in SILP. Related to Figure 2.

(A) Small intestinal lamina propria $CD4^+CD44^+$ T cells were stained for I-A^b $dmLT_{166-174}$ tetramer binding and Foxp3 expression to compare the dmLT-specific $CD4^+$ T cell effector responses between T_{WT} and T_{AKO} mice.

(B) Gated dmLT tetramer$^+$ T cells from representative T_{WT} (black dot plot) and T_{AKO} (red dot plot) SILP were analyzed for expression of T-bet and RORγt.

(C and D) Histograms depicting expression of RORγt (C) and CCR6 (D) in T_{WT} and T_{AKO} dmLT tetramer$^+$ RORγt$^+$ Th17 cells. Geometric mean fluorescence intensities (gMFI) are included in parentheses.

(E) Correlation between RORγt expression (gMFI) and percent $CCR6^+$ oral vaccine-specific Th17 cells.

(F) Representative flow cytometric analysis of SILP-accumulated T_{WT} (black dot plot) and T_{AKO} (red dot plot) 7B8tg cells at 2 weeks post adoptive transfer.

(G) Histogram of RORγt expression in T_{WT} and T_{AKO} RORγt$^+$ 7B8 Th17 cells. gMFI are included in parentheses.

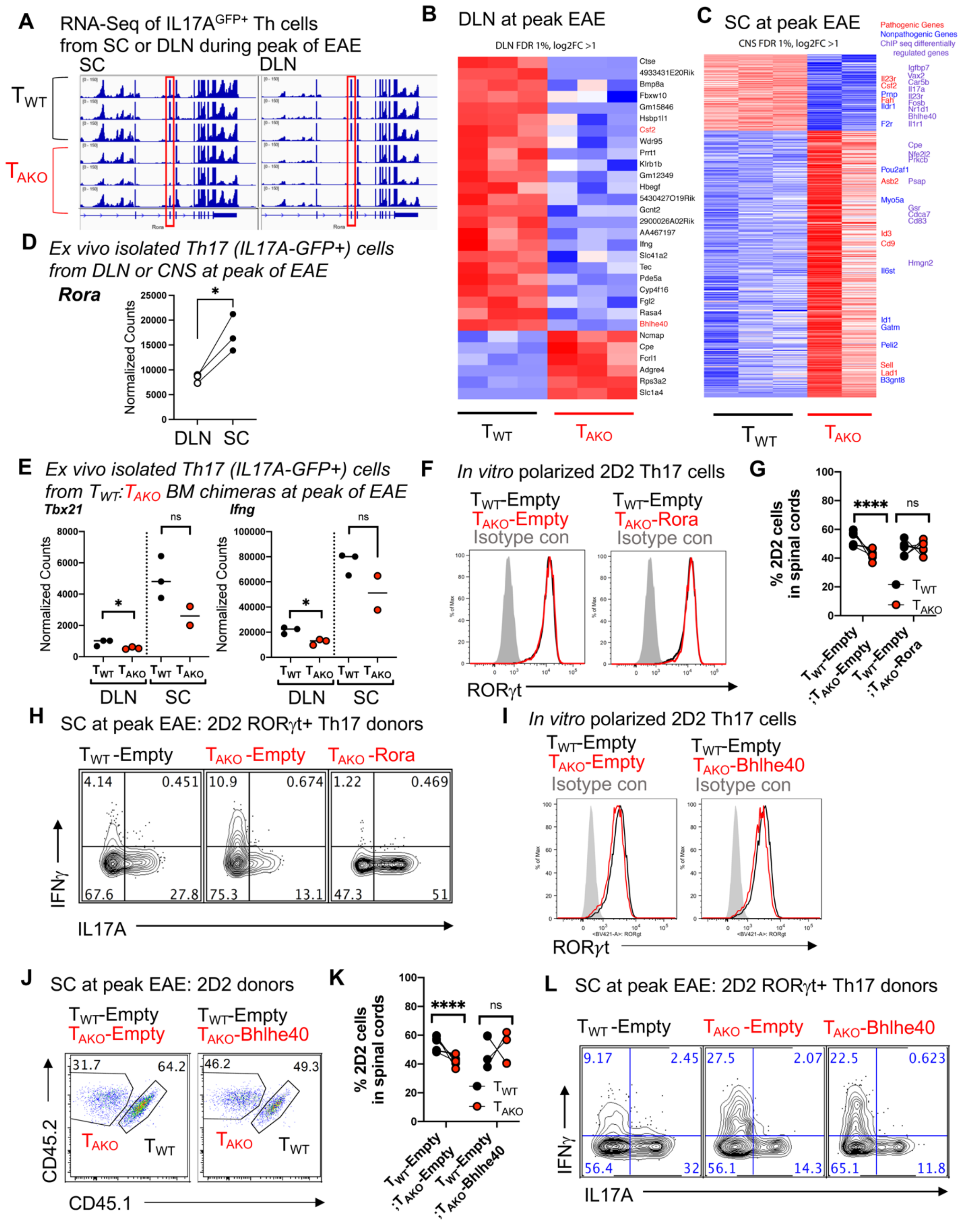

A RNA-Seq of IL17A GFP+ Th cells from SC or DLN during peak of EAE
SC
DLN
TWT
TAKO
B DLN at peak EAE
DLN FDR 1%, log2FC >1
Ctse
4933431E20Rik
Bmp8a
Fbxw10
Gm15846
Hsbp1l1
Csf2
Wdr95
Prrt1
Klrb1b
Gm12349
Hbegf
5430427O19Rik
Gcnt2
2900026A02Rik
AA467197
Ifng
Slc41a2
Tec
Pde5a
Cyp4f16
Fgl2
Rasa4
Bhlhe40
Ncmap
Cpe
Fcrl1
Adgre4
Rps3a2
Slc1a4
TWT
TAKO
C SC at peak EAE
CNS FDR 1%, log2FC >1
Pathogenic Genes
Nonpathogenic Genes
ChIP seq differentially regulated genes
TWT
TAKO
D Ex vivo isolated Th17 (IL17A-GFP+) cells from DLN or CNS at peak of EAE
Rora
Normalized Counts
DLN
SC
E Ex vivo isolated Th17 (IL17A-GFP+) cells from TWT:TAKO BM chimeras at peak of EAE
Tbx21
Ifng
Normalized Counts
DLN
SC
F In vitro polarized 2D2 Th17 cells
TWT-Empty
TAKO-Empty
Isotype con
TWT-Empty
TAKO-Rora
Isotype con
RORγt
G
% 2D2 cells in spinal cords
TWT-Empty ;TAKO-Empty
TWT-Empty ;TAKO-Rora
H SC at peak EAE: 2D2 RORγt+ Th17 donors
TWT -Empty
TAKO -Empty
TAKO -Rora
IFNγ
IL17A
I In vitro polarized 2D2 Th17 cells
TWT-Empty
TAKO-Empty
Isotype con
TWT-Empty
TAKO-Bhlhe40
Isotype con
RORγt
J SC at peak EAE: 2D2 donors
TWT-Empty
TAKO-Empty
TWT-Empty
TAKO-Bhlhe40
CD45.2
CD45.1
K
% 2D2 cells in spinal cords
TWT-Empty ;TAKO-Empty
TWT-Empty ;TAKO-Bhlhe40
L SC at peak EAE: 2D2 RORγt+ Th17 donors
TWT -Empty
TAKO -Empty
TAKO-Bhlhe40
IFNγ
IL17A

Figure S3. Identification of RORα target genes with RNA-Seq analysis and rescue of EAE phenotype with T_{AKO} cells expressing ectopic RORα or its target gene product BHLHE40. Related to Figure 3.

(A) RNA-Seq tracks within *Rora* locus indicating efficient inducible deletion of *Rora* (Exon3) of *Il17a*$^{eGFP+}$ T_{WT} and T_{AKO} Th17 cells from DLN and SC of mixed BM chimera mice at peak of EAE. RNA preparation from sorted *Il17a*$^{eGFP+}$ mice is described in Methods. One SC T_{AKO} Th17 sample contained reads in the deleted region of the *Rora* locus and thus was excluded from analysis; all other T_{AKO} samples were devoid of reads in this region.

(B and C) Clustered heatmap of differentially expressed genes between *Il17a*$^{eGFP+}$ Th17 T_{WT} and T_{AKO} cells from DLN (B) and SC (C) of mixed BM chimera mice at peak of EAE. Color scale is based on z-scores for each gene. Genes listed on the righthand margin are color coded. Blue = non-pathogenic Th17 signature. Red = pathogenic Th17 signature. Purple = Genes associated with RORα ChIP-Seq peaks.

(D) Normalized counts of *Rora* mRNA in *Il17a*$^{eGFP+}$ Th17 cells from the DLN (n = 3) and CNS (n = 3) at peak of EAE.

(E) Normalized counts of Th1-associated mRNAs in T_{WT} and T_{AKO} *Il17a*$^{eGFP+}$ Th17 cells from the DLN (T_{WT} (n = 3) and T_{AKO} (n = 3)) and CNS (T_{WT} (n = 3) and T_{AKO} (n = 2)).

(F-L) Reconstitution of 2D2 T_{AKO} cells with RORα or BHLHE40 and phenotypic analysis in spinal cords during EAE.

(F) Stacked histogram illustrating representative RORγt expression of *in vitro* polarized 2D2tg T_{WT}-Empty and *Rora*-deficient (T_{AKO}-Empty) or -reconstituted (T_{AKO}-Rora) Th17 cells.

(G) Frequency of co-transferred T_{WT} and T_{AKO} donor-derived 2D2tg cells, retrovirally reconstituted with or without *Rora*, in the SC at peak of EAE.

(H) Representative FACS plots displaying IL-17A and IFNγ production of RORγt$^+$ Th17 T_{AKO}-Empty or T_{AKO}-Rora 2D2 cells compared to T_{WT}-Empty upon ex vivo PMA/Ionomycin restimulation.

(I) Stacked histogram illustrating representative RORγt expression of *in vitro* polarized 2D2tg T_{WT}-Empty and *Rora*-deficient (T_{AKO}-Empty) or *Bhlhe40*-overexpressing (T_{AKO}-Bhlhe40) Th17 cells.

(J and K) Representative FACS plots (J) and frequencies (K) of co-transferred T_{WT} and T_{AKO} donor-derived 2D2tg cells, retrovirally transduced with or without *Bhlhe40*, in the SC at peak of EAE.

(L) Representative FACS plots displaying IL-17A and IFNγ production of RORγt$^+$ Th17 T_{AKO}-Empty or T_{AKO}-Bhlhe40 2D2 cells compared to T_{WT}-Empty upon ex vivo PMA/Ionomycin re-stimulation.

Data combined from two experiments. Statistics were calculated using the paired sample T test. ns = not significant, *p < 0.05, **p < 0.01, ***p < 0.001, ****p < 0.0001.

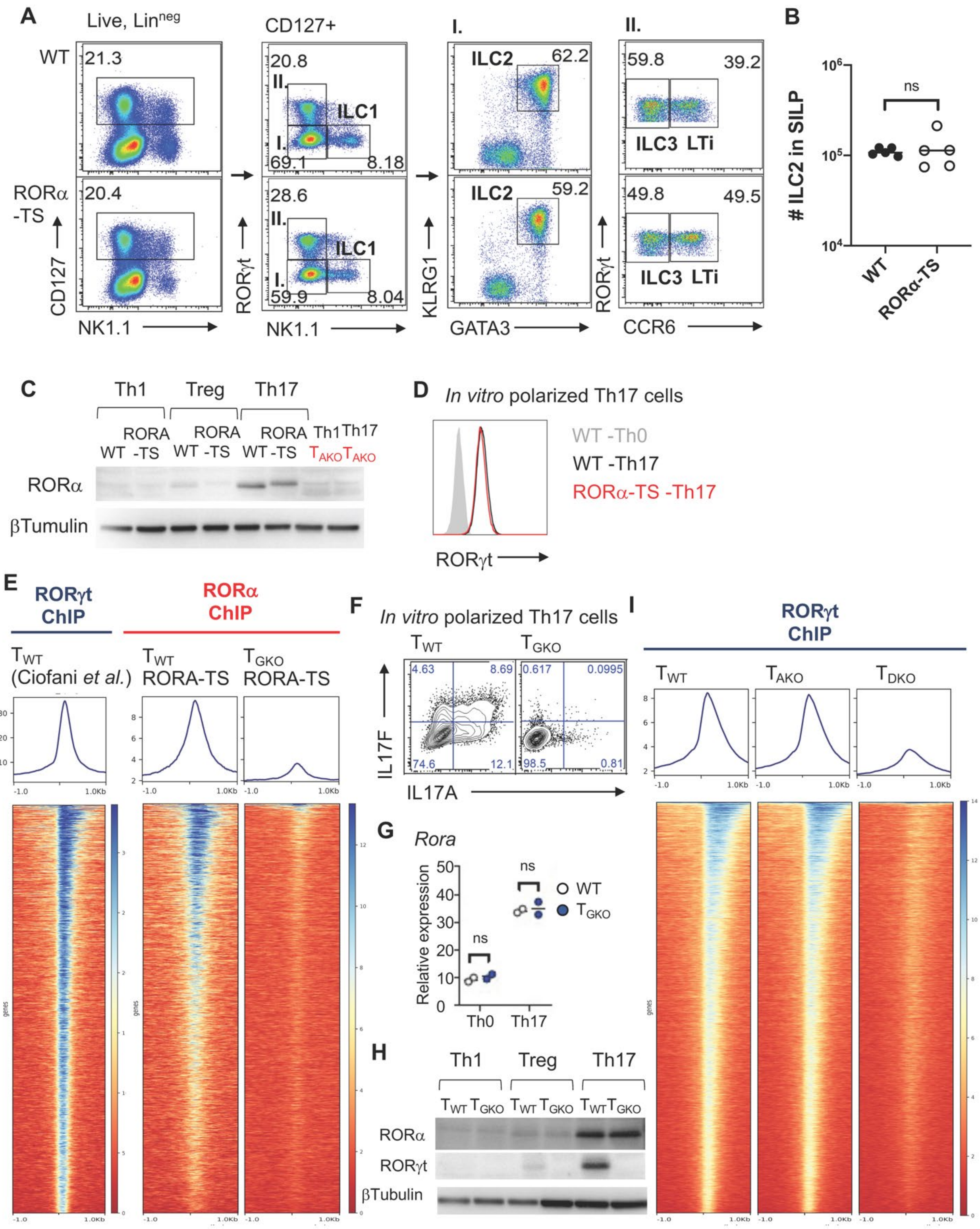

Figure S4. Genomic binding sites of RORα and RORγt are shared in Th17 cells. Related to Figure 4.

(A) Gating strategy to identify innate lymphoid cell (ILC) populations in small intestine lamina propria (SILP) of wild type (WT) and RORA-TS mice. Lineage markers (Lin) include CD3, TCRβ, TCRγδ, CD11b, CD19.

(B) Absolute number of ILC2 (Lin^{neg}, $CD127^{+}$, $ROR\gamma t^{neg}$, $NK1.1^{neg}$, $KLRG1^{+}$, $GATA3^{+}$) in SILP of WT and RORA-TS mice.

(C) Western blot data displaying intact RORα expression of *in vitro* polarized RORA-TS Th17 cells.

(D) Stacked histogram illustrates representative RORγt expression of *in vitro* polarized RORA-TS Th17 cells.

(E) Heatmaps depicting genome-wide RORγt (left) and RORα-TS (middle and right) ChIP-Seq signals of *in vitro* polarized Th17 cells, centered on the summit of RORγt binding sites called on the basis of our earlier dataset (Ciofani et al., 2012). Middle and right alignments compare RORα occupancy in wild-type and RORγt-deficient T cells.

(F) Representative FACS plots displaying IL-17A and IL-17F production of *in vitro* polarized T_{WT} or T_{GKO} Th17 cells.

(G) qPCR result of *Rora* gene expression of in vitro polarized T_{WT} and T_{GKO} cells cultured under Th0 (IL-2) or Th17 (IL-6+TGF-β+IL-23) conditions for 48h.

(H) Immunoblots for RORα and RORγt of in vitro polarized T_{WT} and T_{GKO} cells cultured under Th1 (IL-2+IL-12), Treg (IL-2+TGF-β) or Th17 (IL-6+TGF-β+IL-23) conditions for 48h. β-Tubulin is shown as a loading control.

(I) Heatmaps representing RORγt ChIP-Seq peaks of *in vitro* polarized T_{WT} (left), T_{AKO} (middle) and RORα/RORγt double knock-out (T_{DKO}) (right) Th17 cells.

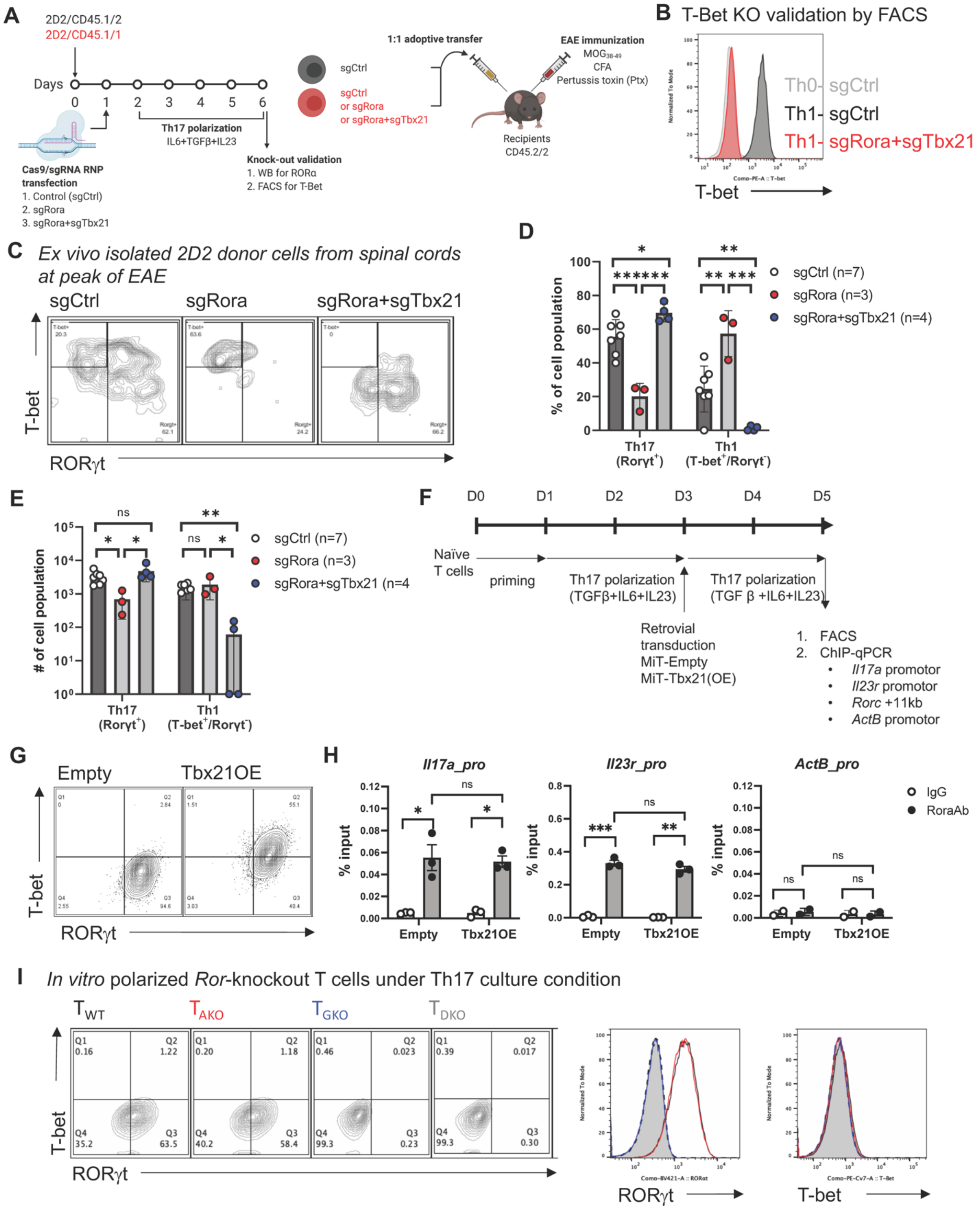

Figure S5. T-bet and RORα regulate RORγt, as well as the Th17 program, via autonomous processes. Related to Figure 4.

(A) Experimental scheme for examining the involvement of RORα and T-bet in the maintenance and/or inhibition of the Th17 program during EAE.

(B) Validation of T-bet deletion by Crispr-mediated genome mutation. Stacked histogram illustrates T-bet expression in control (sgRNA control; sgCtrl) and *Rora*/*Tbx21* double deletion mutant (sgRora+sgTbx21) *in vitro* differentiated 2D2tg Th1 cells.

(C) Representative flow cytometry analysis of RORγt and T-bet expression in control (sgCtrl), Rora single deletion mutant (sgRora) and *Rora*/*Tbx21* double deletion mutant (sgRora+sgTbx21) 2D2tg cells in SC at peak of EAE.

(D and E) Frequency (D) and number (E) of Th17 (RORγt^{+}) or Th1 (T-Bet^{+}/RORγt^{-}) 2D2tg cells in SC at peak of EAE. Summary of 2 experiments, with sgCtrl (n = 7), sgRora (n = 3) and sgRora+sgTbx21 (n = 4) recipients.

(F) Experimental scheme to determine if ectopic T-bet overexpression has an effect on RORα binding to *Il17a* and *Il23r* regulatory elements in committed Th17 cells *in vitro*.

(G) T-bet expression in *in vitro* polarized Th17 cells 2 days after *Tbx21* retroviral transduction.

(H) RORα ChIP qPCR analysis of target loci involved in the "pathogenic" Th17 effector program. Ectopic over-expression (OE) of *Tbx21* in *in vitro* polarized mouse Th17 cells was followed by chromatin immunoprecipitation with rabbit immunoglobulin G (IgG; control) or anti-RORα (RoraAb) and quantitative PCR analysis of binding at the *Il17* promoter, *Il23r* promoter and *ActB* promoter (negative control) (primers are listed in the Table S4). Results were normalized to those of a standardized aliquot of input chromatin. Summary of 3 experiments.

(I) Representative FACS plots(left) and histograms(right) displaying RORγt and T-bet expression of *Ror*-deficient T cells during *in vitro* Th17 polarization. T_{WT}(black)-*CD4*^{Cre}; T_{AKO}(red)-*CD4*^{Cre}*Rora*$^{fl/fl}$; T_{GKO}(blue)-*CD4*^{Cre}*Rorc*$^{fl/fl}$; T_{DKO}(grey)-*CD4*^{Cre}*Rora*$^{fl/fl}$*Rorc*$^{fl/fl}$.

Statistics were calculated using the unpaired sample T test. Error bars denote the mean ± s.e.m. ns = not significant, *p < 0.05, **p < 0.01, ***p < 0.001.

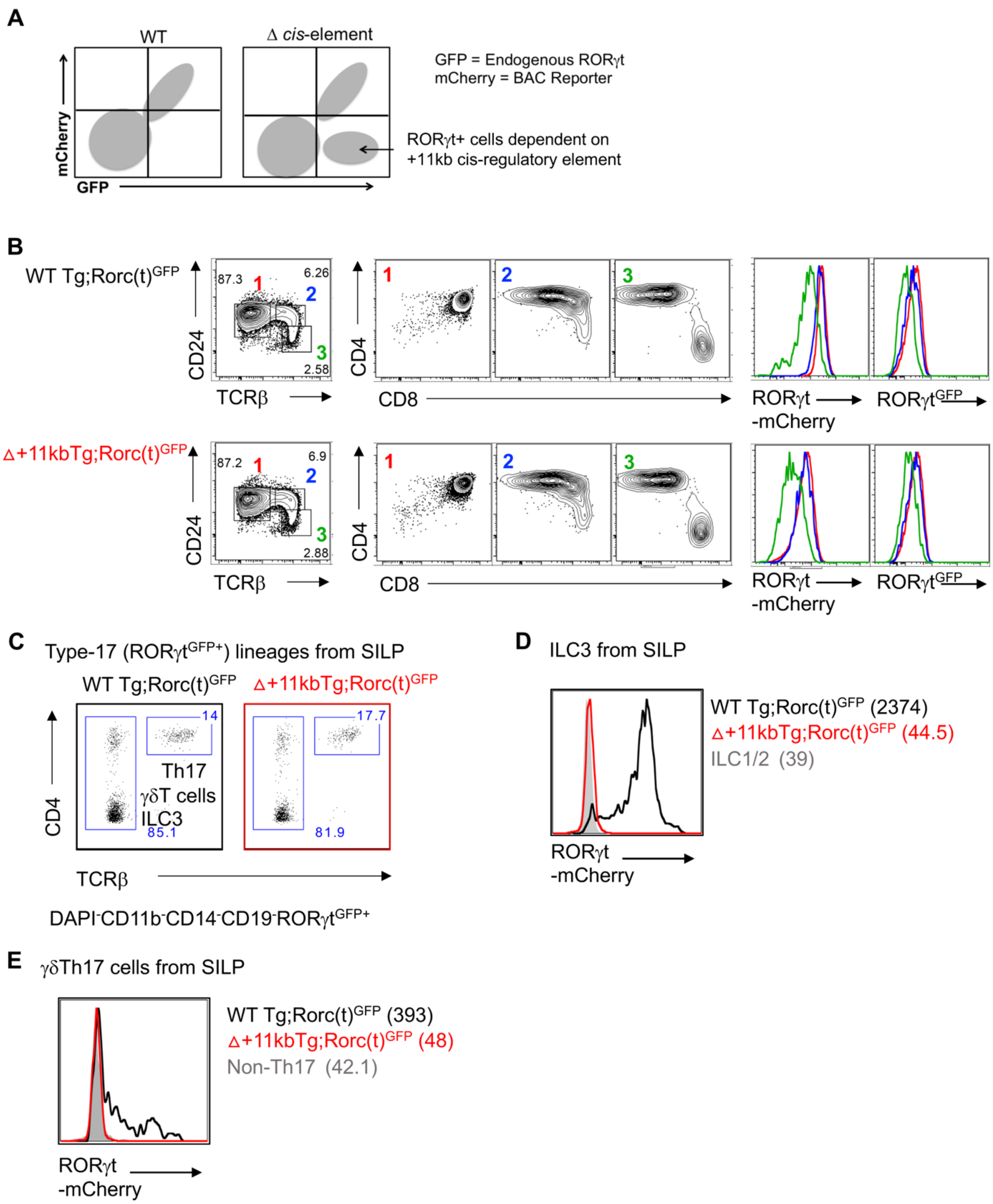

Figure S6. *Rorc(t)* +11kb *cis*-element is required for RORγt expression in Type-17 lymphocytes *in vivo*. Related to Figure 5.

(A) Schematic depicting expression of endogenous and Tg reporter alleles in *Rorc(t)*-mCherry BAC Tg;*Rorc(t)*^{GFP} mice.

(B) Flow cytometry plots depicting gating strategy to capture thymocyte development from DP ($CD4^{+}CD8^{+}$) stage to post-selection stages (left and middle). On the right, mCherry and GFP reporter expression in each color-coded thymocyte subset from indicated Tg mouse line.

(C) Flow cytometry of indicated populations from the SILP in WT and +11kb *cis*-regulatory element mutant Tg (△+11kbTg); *Rorc(t)*^{GFP} mice.

(D and E) mCherry reporter expression of ex vivo isolated ILC3 ($Lin^{neg}ROR\gamma t^{GFP+}$) cells (D) and $\gamma\delta$Th17 ($\gamma\delta TCR^{+}ROR\gamma t^{GFP+}$) cells (E) from SILP of WT Tg; *Rorc(t)*^{GFP} or △+11kbTg;*Rorc(t)*^{GFP} mice. gMFIs are included in parentheses. Representative data of three experiments.

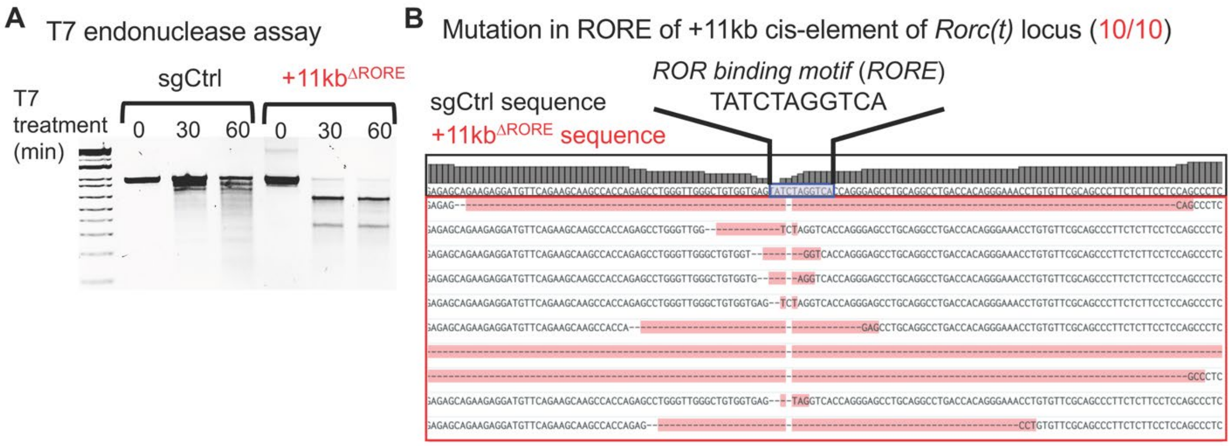

Figure S7. The *Rorc(t)* +11kb *cis*-element is efficiently disrupted by CAS9-RNP in primary T cells. Related to Figure 6 and 7.

(A) Analysis of CAS9/gRNA RNP-mediated targeting efficiency of +11kb *cis*-regulatory element by T7 endonuclease I assay.

(B) Sanger sequencing results displaying *Rorc(t)* +11kb *cis*-regulatory element mutations and deletions of T_{AKO} +11kb$^{\Delta RORE}$ 2D2tg-Th17 cells.

Table S1 - sgRNA and HDR donor sequence, Related to STAR METHODS.

Oligonucleotide	Sequence	Note
Mouse *Rora* target guide	GAGCCAGCTATGCAGATTGA	sgRNA
HDR donor DNA template	CGGAAAAGCTAATGGCATTTAAAGCAATATACCCAG<br>ACATTGTGCGACTCCATTTTCCTCCATTATACAAGGA<br>ATTGTTCACTTCAGAATTTGAGCCAGCTATGCAGATT<br>GACGGAGCAAGCGGATCGGCTTCAGGATCGGCCTC<br>TTGGTCTCACCCACAGTTCGAGAAGGGAGGCGGAT<br>CCGGAGGTGGGTCTGGCGGATCCGCTTGGTCCCAT<br>CCTCAGTTTGAAAAGTAAATGTCGCGCCCGAGCACT<br>TCTAGAACATCTGGAGTACAAACATGAAAGTAAGAG<br>AG	Last *Rora* Exon<br>Linker<br>Strep-tag<br>Stop codons<br>Right homology arm

Table S2 - Primers for GalK recombineering and screening, Related to STAR METHODS.

Primers	Sequence
Galk Rec +11kb F	CAAGGCTCTTCAGCCTCTACTCCAGGCTCTGCTCCAGAAAACCT TTACCA CCTGTTGACAATTAATCATCGGCA
Galk Rec +11kb R	TGTCTATGAGCTTTCTTTTGGGGAGCTTAGGCTCCAGGCTCCTTT CATGC TCAGCACTGTCCTGCTCCTT
+11kb HA F	CAAGGCTCTTCAGCCTCTACTCCAGGCTCTGCTCCAGAAAACCT TTACCA
+11kb HA R	TGTCTATGAGCTTTCTTTTGGGGAGCTTAGGCTCCAGGCTCCTTT CATGC
+11kb screen F	CAAGGCTCTTCAGCCTCTACTC
+11kb screen R	TGTCTATGAGCTTTCTTTTGGGGA

Table S3 - DNA template for *Rorc* deletions, Related to STAR METHODS.

DNA template	Sequence
deletion template+11kb HA F	CAAGGCTCTTCAGCCTCTACTCCAGGCTCTGCTCCAGAAAACCT TTACCAGCATGAAAGGAGCCTGGAGCCTAAGCTCCCCAAAAGA AAGCTCATAGACA
deletion template+11kb HA R	TGTCTATGAGCTTTCTTTTGGGGAGCTTAGGCTCCAGGCTCCTT TCATGCTGGTAAAGGTTTTCTGGAGCAGAGCCTGGAGTAGAGG CTGAAGAGCCTTG

Table S4 – Primers for qPCR analysis, Related to STAR methods

**Primers**	**Sequence**
Rora qRT-PCR forward primer	CATTTGTTCACGAGGCTTTCC
Rora qRT-PCR reverse primer	GTTTTTCCAGTTAGCTTCCTTCATGT
Gapdh qRT-PCR forward primer	AATGTGTCCGTCGTGGATCT
Gapdh qRT-PCR reverse primer	CATCGAAGGTGGAAGAGTGG
Universal 16S qPCR forward primer	ACTCCTACGGGAGGCAGCAGT
Universal 16S qPCR reverse primer	ATTACCGCGGCTGCTGGC
SFB 16S qPCR forward primer	GACGCTGAGGCATGAGAGCAT
SFB 16S qPCR reverse primer	GACGGCACGAATTGTTATTCA
RORα ChIP qPCR analysis for *Il17a* promoter forward primer:	GCAGCAGCTTCAGATATGTCC
RORα ChIP qPCR analysis for *Il17a* promoter reverse primer:	AGATGGGAAGGGCAGAAGTT
RORα ChIP qPCR analysis for *Il23r* promoter forward primer	ACTAGTCATTATGAAATGGACACGAG
RORα ChIP qPCR analysis for *Il23r* promoter reverse primer	TCTTTTCATTTGTTTGCATTACTTTC
RORα ChIP qPCR analysis for *Rorc* +11kb *cis*-regulatory element forward primer	CTGGGTTGGGCTGTGGTGAG
RORα ChIP qPCR analysis for *Rorc* +11kb *cis*-regulatory element reverse primer	GTCTGGGAGGGCTGGAGGAA
RORα ChIP qPCR analysis for *Actb* promoter forward primer	TTTCAAAAGGAGGGGAGAGG
RORα ChIP qPCR analysis for *Actb* promoter reverse primer	TCGAGCCATAAAAGGCAACT
